# Diagnostic and clinical utility of exome sequencing and chromosomal microarray in children with GDD/iD: a meta-analysis

**DOI:** 10.1080/07853890.2025.2609424

**Published:** 2025-12-30

**Authors:** Maliwan Tengsujaritkul, Orawan Louthrenoo, Narueporn Likhitweerawong, Nonglak Boonchooduang, Manit Srisurapanont

**Affiliations:** ^a^ Department of Pediatrics, Faculty of Medicine, Chiang Mai University, Chiang Mai, Thailand; ^b^ Department of Psychiatry, Faculty of Medicine, Chiang Mai University, Chiang Mai, Thailand

**Keywords:** Diagnostic yield, exome sequencing, genetic testing, global developmental delay, intellectual disability

## Abstract

**Background:**

Global developmental delay/intellectual disability (GDD/ID) is among the most common neurodevelopmental disorders, with up to half of cases are attributed to genetic factors. Chromosome microarray (CMA) has traditionally been the primary genetic test for idiopathic GDD/ID. However, whole exome sequencing (WES) and whole genome sequencing (WGS) have recently emerged, substantially increasing diagnostic yields in these populations.

**Methods:**

We conducted a comprehensive literature search of PubMed, Scopus, EMBASE, and the Cochrane Library from inception to April 29, 2025. Studies reporting the diagnostic utility of these tests in children with GDD/ID were included and analyzed.

**Results:**

A total of 102 studies, comprising 55,752 children, were reviewed. The pooled diagnostic yield of WES was 0.37 (95% CI: 0.33–0.41; I^2^ = 93%), significantly higher than that of CMA at 0.19 (95% CI: 0.16–0.21; I^2^ = 95%). Subgroup analyses showed that WES yielded significantly higher diagnostic rates than CMA in both same-sample comparisons (OR = 2.27, 95% CI: 1.08–4.78) and different-sample comparisons (OR = 1.65, 95% CI: 1.15–2.37). Only one study evaluated WGS, reporting a diagnostic yield of 0.27. Meta-regression revealed a significant association between CMA diagnostic yield and the proportion of male participants (*p* < 0.01), but not with WES. No significant difference in diagnostic utility was observed between isolated GDD/ID and GDD/ID with comorbidities.

**Conclusion:**

In children with unexplained GDD/ID, WES demonstrates superior diagnostic and clinical utility compared to CMA. Incorporating WES as a first-line investigation in the diagnostic evaluation of GDD/ID may be warranted.

## KEY MESSAGES

- The pooled diagnostic yield of whole exome sequencing (WES) was 0.37 (95% CI: 0.33–0.41), significantly higher than that of chromosomal microarray (CMA) at 0.19 (95% CI: 0.16–0.21) in children with global developmental delay/intellectual disability (GDD/ID).
- No significant difference in diagnostic utility was observed between isolated GDD/ID and GDD/ID with comorbidities.
- WES may be warranted as a first-line investigation in the diagnostic evaluation of GDD/ID.

## Introduction

Global developmental delay (GDD) and intellectual disability (ID) are neurodevelopmental disorders affecting approximately 1-3% in children [1,2]. GDD is characterized by delays in achieving developmental milestones relative to age expectations, involving significant impairment in two or more domains—gross or fine motor skills, speech/language, cognitive functioning, social/personal development, and activities of daily living [1]. The term is typically applied to children under the age of 5, for whom standardized IQ testing is not yet reliable.

According to the Diagnostic and Statistical Manual of Mental Disorders, 5th Edition (DSM-5) criteria, ID is defined as deficits in both intellectual and adaptive functioning, affecting conceptual, social, and practical domains, with onset during the developmental period [3]. The diagnosis is generally reserved for older children when formal cognitive assessments can be reliably performed. GDD and ID are closely related, as most children initially diagnosed with GDD later meet criteria for ID [4], however, this progression is not always observed [5].

The exact etiology of GDD/ID is often unknown [6], but may include neurological, metabolic, and genetic causes. Genetic disorders account for up to 50% of identified cases [4,7–9]. Although many cases of GDD/ID appear isolated and yield negative genetic test results, growing evidence suggests that approximately 20-80% of affected individuals may have an identifiable genetic cause. Diagnostic yield is higher in severe cases—such as those with dysmorphic features, congenital anomalies, intractable seizures, or neurological deficits—than in milder presentations [10,11].

GDD/ID are most often associated with genetic alterations, including insertions/deletions, single nucleotide variations, and copy number variations [4]. Broad genetic categories include chromosomal, monogenic, oligo/polygenic, and imprinting-related disorders. Commonly identified syndromes in this population include Down syndrome, 22q11.2 deletion syndrome, Prader-Willi syndrome, Angelman syndrome, Williams syndrome, and Fragile X syndrome [10].

Identifying an underlying genetic etiology is important not only for diagnostic clarification but also for providing affected individuals and their families with a clearer understanding of the condition’s prognosis and available treatment options. This knowledge can guide expectations for developmental outcomes, anticipate potential health complications, and support informed decision-making regarding family planning and genetic counseling [10,12].

Most guidelines recommend genetic testing for all individuals with GDD/ID to identify potential underlying genetic causes. When a specific syndromic pattern is recognized, initial testing can follow a phenotype-driven approach—such as targeted testing for specific syndromes using methods like fluorescence *in situ* hybridization (FISH) or karyotyping. In contrast, nonsyndromic GDD/ID is typically evaluated using broader genetic tests with higher diagnostic yield [10].

Chromosomal microarray (CMA) is recommended as a first-tier diagnostic test for all individuals with GDD/ID, alongside Fragile X testing, due to its ability to detect pathogenic copy number variants (CNVs), deletions or duplications of DNA segments, rather than karyotyping, in a significant proportion of cases [1]. CMA has a reported diagnostic yield rate to detect genetic underlying conditions in patients with GDD/ID, ranging from 12 to 20% [2,13,14]. However, the American College of Medical Genetics and Genomics (ACMG) and American Academy of Pediatrics (AAP) have recently suggested that whole exome sequencing or whole genome sequencing (WES/WGS) may be considered as first-tier tests in patients with GDD/ID due to their higher diagnostic yield and cost-effectiveness, especially when performed early in the evaluation process [15].

WGS has the advantage of detecting CNVs, structural rearrangements, and nearly the entire whole genome—including intronic and noncoding regions not covered by WES, which targets only exon regions. Consequently, WGS typically achieves a higher diagnostic yield than WES, which has been reported to range from 27% to 45% [10,16,17].

Despite these advantages, debate continues over which test should be prioritized, considering factors such as cost-effectiveness, turnaround time, and the potential for incidental or uncertain findings. Accessibility also plays a role; in some healthcare settings, CMA remains more widely available and less expensive than WES/WGS. Nonetheless, the superior diagnostic performance of WES/WGS has led some experts and institutions to recommend it as a first-tier option in selected cases.

In this context, our meta-analysis aims to systematically review and synthesize available evidence to estimate the diagnostic yield of CMA and WES in individuals with GDD/ID, and to directly compare these rates. We also conducted subgroup and meta-regression analyses to explore potential moderators of diagnostic yield.

## Material and methods

### Protocol and registration

This meta-analysis followed the Preferred Reporting Items for Systematic Reviews and Meta-Analyses (PRISMA) 2020 guidelines [18]. The protocol was registered with the Open Science Framework on May 14, 2025 (available at: https://osf.io/u6wsv/). The study was reviewed and granted an exemption by the Ethics Committee of the Faculty of Medicine, Chiang Mai University, Thailand (Study code: PED-2568-0602).

### Data sources and record identification

We systematically searched PubMed, Scopus, EMBASE, and Cochrane Library databases from inception to April 29, 2025. Details of the search strategy are provided in Supplementary Table 1 of the Supplementary File.

### Eligibility criteria, information sources, and searches

We included prospective or retrospective studies regardless of study design if they met the following criteria:

1. The study population included children or adolescents diagnosed with global developmental delay (GDD), intellectual disability (ID), or other neurodevelopmental disorders. Studies focusing exclusively on adults or with a minimum age threshold of >18 years were excluded.
2. The study reported diagnostic utility from genetic testing methods, including chromosomal microarray (CMA), whole exome sequencing (WES), or whole genome sequencing (WGS).
3. Studies involving isolated GDD/ID or GDD/ID with comorbidities (e.g. congenital anomalies, neurological abnormalities, or autism spectrum disorder) were included.
4. Full-text articles or conference proceedings with sufficient data relevant to the study objectives were available.

We excluded studies that:

1. Focused primarily on autism spectrum disorder, congenital anomalies, or unspecified neurodevelopmental disorders without a clear diagnosis of GDD/ID.
2. Reported sequential genetic testing (e.g. WES following a negative CMA result).
3. Did not report diagnostic or clinical utility.

**Figure 1.**
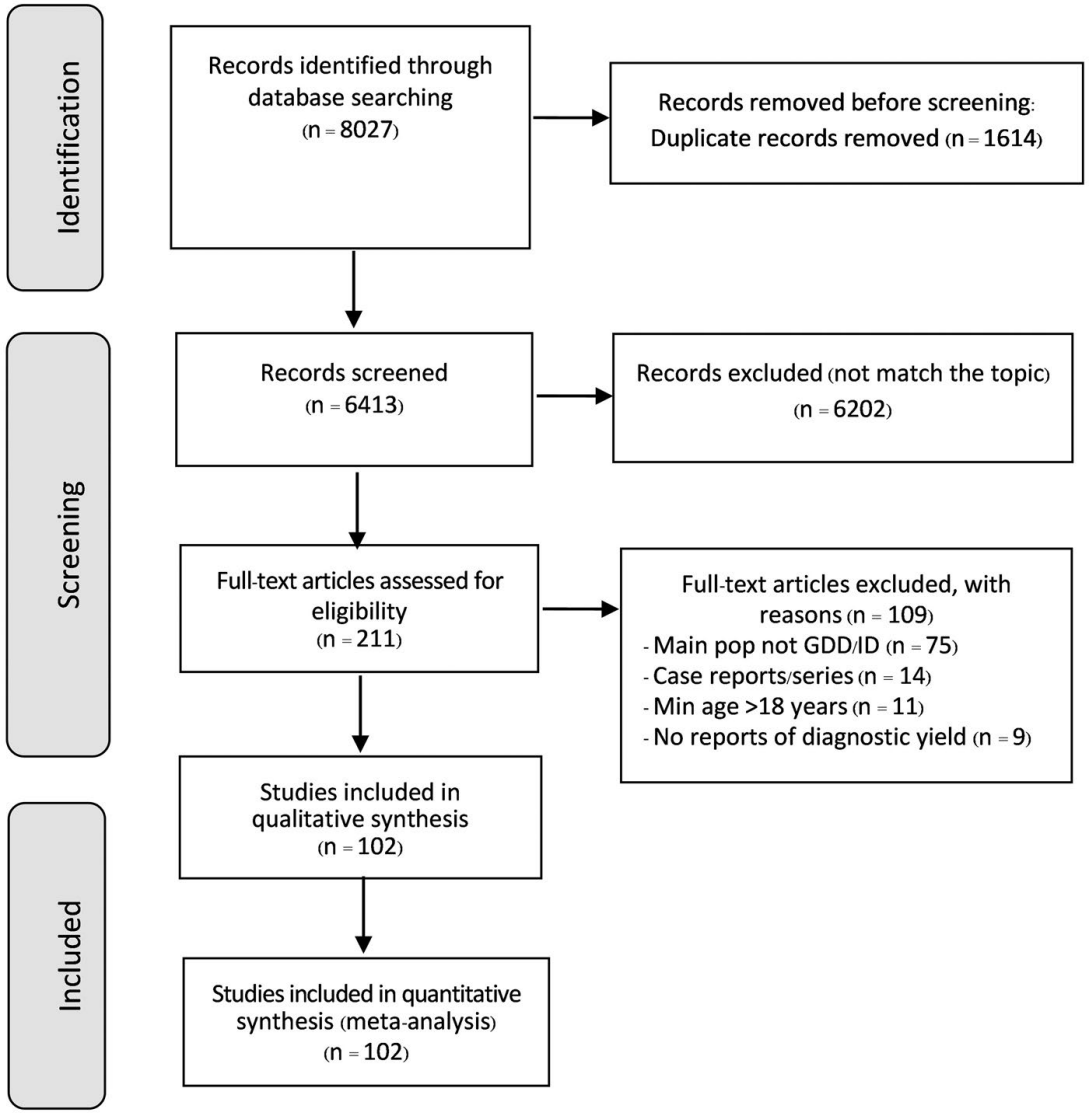
PRISMA flow of database searches, study selection, and study inclusion in the proportion meta-analysis of CMA, WES, and WGS diagnostic rates.

The literature search was conducted independently by two authors (MT and OL), using the following search terms, as in Supplementary Table 1 supplementary file. Details of the study search and selection process are presented in the PRISMA flow chart in Figure 1.

### Study selection and data extraction

After duplicate removal, titles and abstracts were screened for eligibility. Full texts were reviewed to determine final inclusion. Studies not published in English or with non-English abstracts were excluded.

Data extraction was performed independently by two reviewers (MT and OL). Extracted data included:

- Study year and first author
- Participant characteristics (age, sex, number)
- Type of genetic testing and its diagnostic yield

When available, comorbidity data were extracted for subgroup analysis and divided into three subgroups as follows: not specified, with comorbidity, and without comorbidity. Discrepancies were resolved by a third reviewer through discussion.

### Quality assessment

The quality of studies was evaluated independently by two reviewers using the tool for assessing risk of bias in prevalence studies which was modified for this descriptive study [19]. This tool evaluates:

- External validity: representativeness of the target population, sampling method, and non-response bias
- Internal validity: data collection method, case definition, reliability of measurement tools, study period, and appropriateness of the numerator and denominator

Assessment results are summarized in Supplementary Table 2. Any disagreements were resolved through consensus.

### Statistical analysis

The primary effect measure was the diagnostic yield, defined as the proportion of participants receiving a molecular diagnosis by CMA, WES, or WGS among the total tested. Diagnostic yields were logit-transformed and synthesized using a random-intercept logistic regression (one-step generalized linear mixed model, GLMM [20]. In this proportional meta-analysis, we applied a random-effects model and estimated between-study variance using the maximum likelihood method.

For studies reporting more than one test modality, diagnostic yields were compared using a random-effects logistic regression model with fixed effects for study to account for within-study pairing. Odds ratios (ORs) and 95% confidence intervals (CIs) were estimated *via* maximum likelihood methods.

Between-study heterogeneity was quantified using the I^2^ statistics, values ≥75% considered to indicate substantial heterogeneity [21]. Publication bias was evaluated using funnel plots, Egger’s test for funnel plot asymmetry, and the trim-and-fill method.

Sensitivity analyses excluded studies with a high risk of bias. Subgroup analyses were performed based on comorbidity status—(i) unspecified, ii) present, and iii) absent—and differences among subgroups were assessed using Cochran’s Q test. Meta-regression analyses were conducted with mean age, proportion of male participants, and publication year as covariates within a logistic regression framework. We did not assess certainty of evidence due to limitations of the current Grading of Recommendations Assessment, Development, and Evaluation (GRADE) approach for meta-analyses of observational studies [22].

## Results

### Study selection, characteristics, and risk of bias

Database searches retrieved 8,027 records, as presented in Figure 1. After removing duplicates and nonrelevant articles, a total of 102 studies conducted across 37 countries were included in the meta-analysis (Supplementary Table 2). Table 1 summarizes the characteristics of the included studies, including authors, publication year, country, study population, participant age and sex, type of genetic testing, number of participants, and diagnostic utility of each test.

**Table 1.** Characteristics of studies reporting diagnostic or clinical utility of CMA, WES, and WGS.

Author	Study year	Country	Population	Age range	Mean/median age (yr)	% Male	Genetic testing	N	Diagnostic yield (n)
Abarca-Barriga [3]	2025	Peru	ID		6.3	57.3	ES	134	38
Abe-Hatano [23]	2021	Japan	ID			57.8	GS	45	12
Akkus [26]	2024	Turkey	DD w/wo ASD, comorbid			57.1	CMA	1227	135
Akter [27]	2023	Bangladesh	NDD w/wo ASD, epilepsy/seizure, hypotonia, etc.			68.9	CMA	212	26
Akter [28]	2025	Bangladesh	NDD w/wo comorbid	0–18 yr		67.7	CMA	247	42
			NDD w/wo comorbid	0–18 yr		68.7	ES	127	38
Arican [29]	2018	Turkey	DD/ID				CMA	210	26
Asadollahi [30]	2014	Switzerland	NDD				CMA	714	58
Ballesta-Martínez [31]	2023	Spain	ID		4.9		ES	188	64
Bartnik [32]	2014	Poland	DD/ID w/wo dysmorphic features, etc.				CMA	256	51
Battaglia [33]	2013	Italy	DD/ID w/wo ASD, dysmorphic features	5 mo-19 yr		63.9	CMA	349	77
Bhatia [34]	2021	Singapore	DD/ID				ES	82	35
Boyarchuk [35]	2024	Korea	DD w/wo ASD, comorbid	0–18 yr	6.3	67.8	ES	90	21
Brea-Fernández [36]	2022	USA	ID	4–63 yr	29.9	53.9	ES	254	64
Çebi [37]	2020	Turkey	DD w/wo MCA	1 mo-16 yr		58.6	CMA	237	36
Cham [38]	2017	Singapore	DD/ID w/wo comorbid				CMA	510	160
Chaves [39]	2019	Brazil	NDD		9.5	61.9	CMA	420	75
Chaves [40]	2024	Brazil	ID w/wo ASD and comorbid	0–55 yr	10.0	61	CMA	1012	170
Chen [41]	2021	China					CMA	120	20
Choucair [42]	2015	Lebanon	DD/ID w/wo congenital anomalies				CMA	149	23
Cooper [43]	2011	USA + Canada	DD/ID				CMA	15767	4052
de Ligt [44]	2012	Netherlands	ID			47.0	ES	100	16
de Souza [45]	2019	Brazil	DD/ID	8 mo-30 yr		49.2	CMA	63	20
Di Biagio [46]	2023	Italy	DD/ID w/wo comorbid				CMA	270	25
Di Gregorio [47]	2017	Italy	DD/ID				CMA	1015	111
Dingemans [48]	2022	Netherlands	ID/NDD		5.7	61.8	ES	1663	436
Dong [49]	2020	China	DD	1–16 yr		60.6	ES	1090	459
Ezugha [50]	2010	US	NDD		5.7	54.9	CMA	82	20
Gao [51]	2019	China	NDD		2.2	61.1	CMA	54	9
			NDD		2.2	61.1	ES	54	25
Ghalamkari [52]	2025	Iran	ID w/wo comorbid		8.0	57.6	ES	99	40
Girirajan [53]	2011	US	ID w/wo MCA		15.0	63.8	CMA	431	69
Gürkan [54]	2020	Turkey	DD/ID		8.5	65.0	CMA	123	20
Henderson [55]	2014	US	NDD w/wo MCA dysmorphic features		4.7		CMA	1780	227
Hu [56]	2019	China	DD/ID	3 mo-17 yr			CMA	332	60
Hu [57]	2017	China	DD/ID				CMA	92	18
Ilic [58]	2024	Spain	DD/ID		2.7	59.0	ES	100	66
Jo [59]	2024	Korea	DD/ID		1.6	51.2	ES	41	20
Kamath [60]	2022	India	DD/ID		2.7	59.7	CMA	67	17
Kanivets [61]	2017	Russia	DD/ID w/wo comorbid				CMA	3299	565
Khalaf [62]	2024	United Arab Emirates	NDD				ES	405	231
Kim [63]	2018	Korea	DD/ID		5.4	52.0	CMA	50	29
Kim [64]	2023	Korea	ID		4.3	71.4	CMA	154	22
			ID		4.3	71.4	ES	154	30
Kim [65]	2019	Korea	NDD				ES	108	45
Kipkemoi [66]	2023	South Africa + Kenya	NDD				ES	99	22
Lai [67]	2024	China	DD, ID w/wo comorbid	0–17 yr	3.1	59	ES	144	61
Lamilla [68]	2025	USA	NDD w/wo comorbid	0–50 yr	5.0	67	ES	1028	190
Lan [69]	2025	China	NDD w/wo comorbid	0–17 yr	3.8	66.1	ES	1106	510
Lee [70]	2019	Taiwan	DD/ID		7.6	56.5	CMA	177	57
Lee [71]	2018	Korea	DD/ID	7 mo-25 yr		53.2	CMA	649	110
Lee [72]	2017	Korea	DD w comorbid		2.4		CMA	27	4
Levchenko [73]	2022	Russia	ID	6 mo-65 yr			CMA	91	16
			ID	6 mo-65 yr			ES	171	33
Li [74]	2024	China	DD/ID w/wo comorbid		3.8	59.5	ES	173	86
Liu [75]	2022	China	DD/ID			58.6	CMA	251	79
Liu [76]	2021	China	DD		1.4	54.3	ES	94	46
Ma [77]	2024	China	DD/ID		2.6	64.4	ES	225	96
Masri [17]	2023	Jordan	DD/ID				ES	154	69
Miclea [78]	2022	Romania	DD/ID		11.7	51.9	CMA	189	35
Monroe [79]	2016	Netherlands	ID		3.0		ES	17	5
Najafi [80]	2019	Iran	DD/ID				ES	100	49
Neuhann [81]	2021	Germany	GDD/ID and/or ASD				ES	97	31
Newman [82]	2016	USA	syndromic ID/DD				ES	806	205
Nicholl [83]	2014	Australia	DD/ID w/wo ass features				CMA	1066	147
Nouri [84]	2021	Iran	unexplained DD/ID and epilepsy	38 d-15 yr	6.2	64.0	ES	61	36
Nowakowska [85]	2008	Poland	ID w dysmorphic features				CMA	91	19
Oğuz [86]	2021	Turkey	DD/ID wo abn (non-synd)	2 mo-39 yr		61.6	CMA	302	33
Palmer [87]	2014	Australia	ID of unk etio				CMA	67	21
Palmieri [46]	2023	Italy	non synd GDD/ID				ES	30	9
Pati [88]	2015	USA	ID w epilepsy + w cong anomaly	2 mo-10 yr		63.0	CMA	19	5
Pereira [89]	2014	Brazil	ID w/wo cong anomalies	2–25 yr		40.0	CMA	15	9
Pérez-Granero [61]	2017	Spain	ID/DD w dysmorphic features				CMA	641	110
Peycheva [90]	2018	Bulgaria	DD/ID w epilepsy	1–22 yr		54.4	CMA	92	14
Postma [24]	2024	Canada	GDD/ID w/wo comorbid	1–18 yr	5.4	72.6	CMA	244	16
			GDD/ID w/wo comorbid	1–18 yr	5.4	72.6	ES	129	13
Pranav Chand [91]	2023	India	NDD w/wo ass features	1–18 yr		58.56	ES	403	127
Preiksaitiene [92]	2016	Lithuania	DD/ID w/wo syndrome				CMA	66	15
Preiksaitiene [93]	2014	Lithuania	DD/ID w/wo syndrome			70.1	CMA	211	29
Qian [94]	2025	China	DD/ID w/wo comorbid				ES	187	75
Quintela [95]	2017	Spain	DD/ID w/wo another condition	3 mo-18 yr		60.4	CMA	573	64
Rauch [96]	2012	Germany	severe non-syndromic ID			37.3	ES	51	20
Rump [97]	2016	Netherlands	ID w microcephaly	0–57 yr	10.0	45.7	ES	35	10
Sánchez Suárez [25] (a) Sánchez Suárez (b)	2024	Spain	ID w/wo comorbid	9 mo-16 yr	3.6/6.0	72.16	ES	67	14
			ID w ASD					47	6
Sandal [98]	2024	India	mod-severe-profound ID	2.5 mo-37.3 yr	4.5	61.54	ES	227	121
Sansovic [99]	2017	Croatia	DD/ID w/wo dys, ASD, cong malf	1 mo-25 yr	7.0		CMA	337	73
Seo [100]	2022	Korea	mild to severe DD/ID				ES	1065	402
Sharma [14]	2016	India	DD/ID w/wo MCA	3 mo-18 yr	4.3	60.4	CMA	106	15
Shchubelka [101]	2024	Ukrain	DD/ID w/wo comorbid	1–18 yr	7.0	60.9	ES	37	22
Shin [102]	2015	Korea	DD/ID w/wo dysmorphic				CMA	96	15
Stojanovic [103]	2020	Serbia	DD/ID w/wo add clinical				ES	88	49
Taskiran [104]	2021	Turkey	non-synd ID w epilepsy, ASD			51.7	ES	59	29
Valaparambil [105]	2025	India	NDDs w/wo comorbid		5.8	53.0	ES	78	32
Valentino [106]	2021	Italy	ID w clinical, comorbid				ES	200	43
Vrijenhoek [107]	2018	Netherlands	syndromic ID				ES	370	128
Wayhelova [108]	2024	Czech Republic	NDDs and associated MCAs	0–34 yr	6.0/7.0	60.0	ES	90	44
Weiss [109]	2021	Israel	DD and DD with epi/anomalies				ES	175	85
Wu [110]	2023	China	DD/ID w/wo ASD, epilepsy, anomalies	3 mo–10 yr	14.5 mo	62.3	CMA	122	46
Wu [111]	2025	China	GDD/ID w/wo comorbid (ASD)				ES	1457	536
Wu [112]	2024	China	GDD/ID w 1 or more comorbid				ES	163	82
Wu [113]	2021	China	DD/ID				CMA	327	130
Xiang [114]	2021	China	DD/ID w/wo add clinical	2–9 yr	5.6	88.2	ES	17	10
Xiao [115]	2018	China	DD/ID w/wo add anomalies				ES	33	19
Xu [116]	2024	China	DD/ID w/wo comorbid	1–18 yr	3.3	64.3	ES	280	94
Yamamoto [117]	2019	Japan	DD/ID or ASD		4.0	60.9	ES	133	39
Yuan [118] (a) Yuan (b)	2021	China	DD/ID wo synd w other comorbid				CMA	2688	568
			DD/ID w synd					3407	971
Zhang [4]	2024	China	DD w/wo add clinical	1–5 yr	2.1	60.0	ES	434	263

CMA, chromosomal microarray; ES, exome sequencing; GS, genome sequencing; ID, intellectual disability; DD, developmental delay; ASD, autism spectrum disorder, NDD, neurodevelopmental disorders, GDD, global developmental delay, w/wo, with or without; yr, year; mo, month; MCA, multiple congenital anomalies.

In total, the review synthesized data from 102 studies involving 55,752 participants. The primary genetic tests evaluated were CMA and WES. Fifty-three studies (*n* = 41,096) reported on CMA, and 55 studies (*n* = 14,819) reported on WES. A small subset of five studies (*n* = 635) assessed both methods, with two of these testing the same cohort of 208 patients. Only one study reported WGS testing in 45 children with a diagnostic yield of 26.67% [23]. The included studies were published between 2011 and 2025.

Across studies, the proportion of male participants were higher than female participants, with a median of 60.6% (interquartile range [IQR]: 56.5% − 64.4%). The median age of participants was 4.90 years (IQR: 3.23–6.32 years). Common comorbidity included congenital anomalies and autism spectrum disorders. Variation in participant selection and study settings contributed to the high heterogeneity observed in the analyses.

Methodological quality was assessed using the Tool for Assessing Risk of Bias in Prevalence Studies (Supplementary Table 3). Of the studies included, 28 were categorized as high risk, 24 as moderate risk, and 50 as low risk.

### Results of individual studies and syntheses

#### Diagnostic utility of CMA

**Figure 2.**
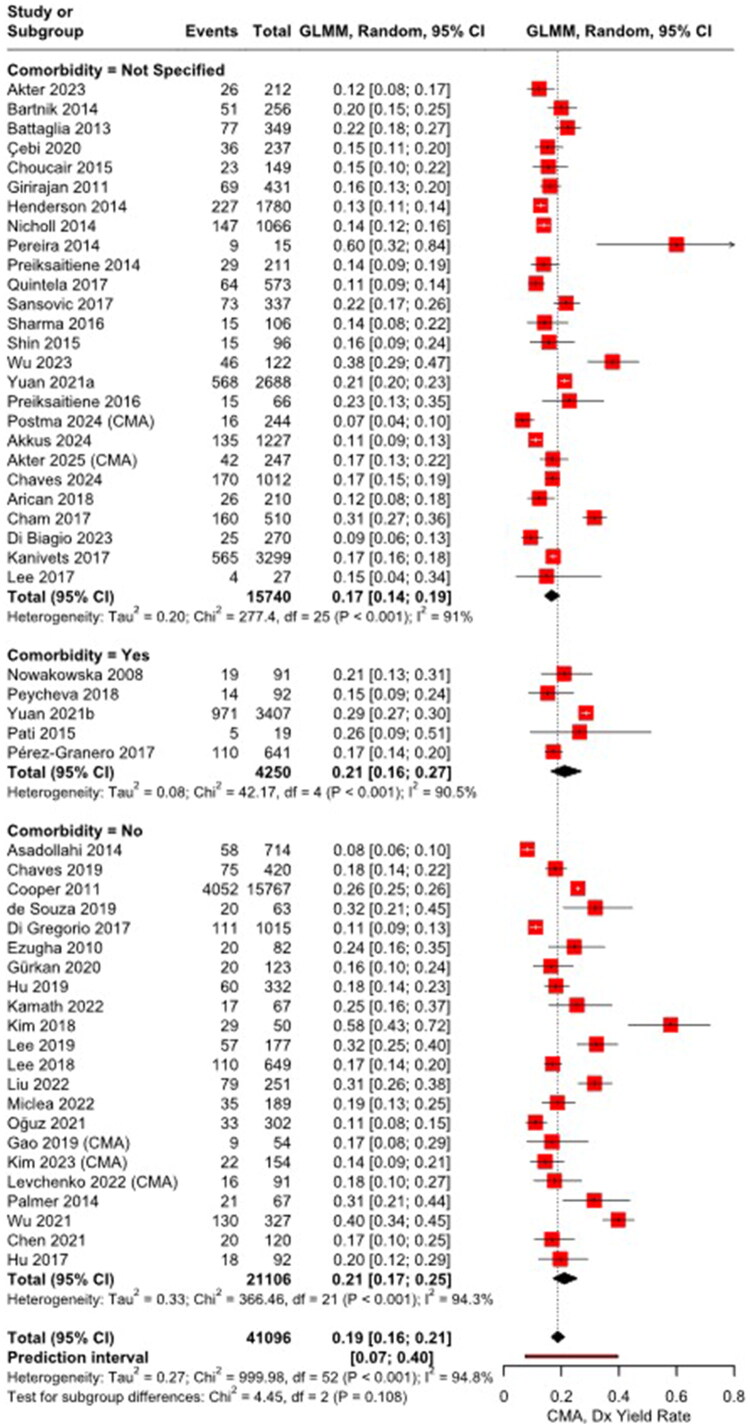
Forest plot of the proportion meta-analysis of CMA diagnostic yields. CMA: chromosomal microarray.

Among 53 studies (*n* = 41,096), Pereira (2014) and Kim (2018) reported relatively high diagnostic yields of 60% (95% CI: 32–84%) and 58% (95% CI: 43–72%), respectively. The meta-analysis of CMA testing showed a pooled diagnostic yield of 19% (95% CI: 16–21%) (Figure 2). Subgroup yields were similar: 17% (95% CI: 14–19%) in cohorts with unspecified comorbidity status, 21% (95% CI: 16–27%) in cohorts with comorbidity, and 21% (95% CI: 17–25%) in cohorts without comorbidity. Differences between subgroups were not statistically significant (*p* = 0.11). Statistical heterogeneity was high within each subgroup (I^2^ = 91–95%) and overall (I^2^ = 94.8%).

Supplementary Figure 1 shows evidence of significant publication bias (Egger’s regression intercept β = 2.33, SE = 0.78, *p* < 0.01). The trim-and-fill method added 16 hypothetical studies to the 53 observed, yielding an adjusted pooled estimate of 23.5% (95% CI: 20.6–26.7%), suggesting that some higher-yield studies may be missing. After excluding 14 studies with a high risk of bias, the sensitivity analysis of the remaining 39 studies produced a consistent pooled yield of 19% (95% CI: 16–22%) (Supplementary Figure 2).

Meta-regression revealed a significant association between CMA diagnostic yield and the proportion of male participants (*n* = 30, coefficient = −0.06, *p* < 0.01), but not with mean age (*n* = 17, coefficient = −0.03, *p* = 0.53) or publication year (*n* = 53, coefficient = −0.01, *p* = 0.49) (Supplementary Figure 4).

#### Diagnostic utility of WES

**Figure 3.**
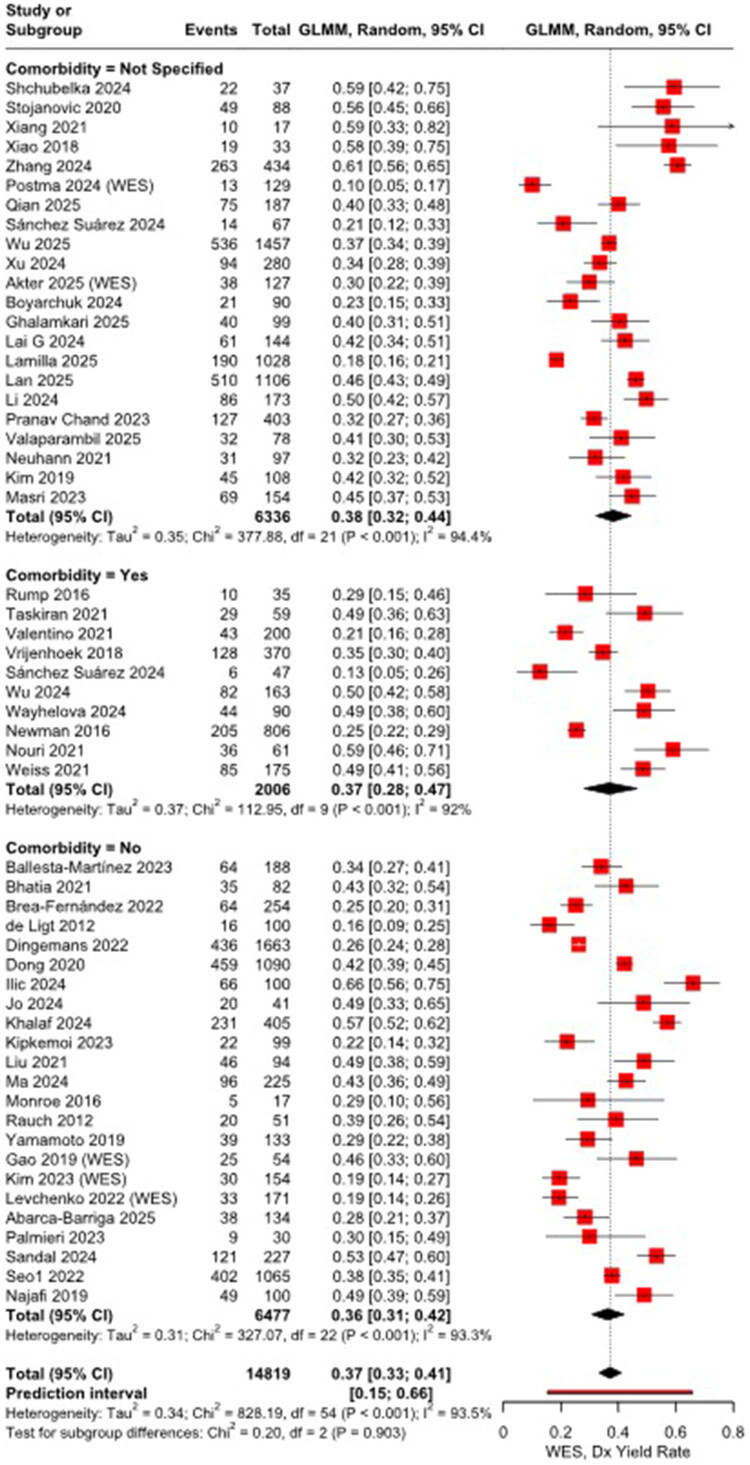
Forest plot of the proportion meta-analysis of WES diagnostic yields. WES, whole exome sequencing.

Among 55 studies (*n* = 14,819), Postma, et al. [24] and Sanchez Suarez, et al. [25] reported relatively low diagnostic yields of 10% (95% CI: 5–17%) and 13% (95% CI: 5–26%), respectively. The meta-analysis of WES testing showed a pooled diagnostic yield of 37% (95% CI: 33–41%) (Figure 3). Subgroup yields were similar: 38% (95% CI: 32–44%) in cohorts with unspecified comorbidity status, 37% (95% CI: 28–47%) in cohorts with comorbidity, and 36% (95% CI: 31–42%) in cohorts without comorbidity. Differences between subgroups were not statistically significant (*p* = 0.90). Statistical heterogeneity was high within each subgroup (I^2^ = 92–94%) and overall (I^2^ = 93.5%).

Supplementary Figure 1 shows no significant publication bias (Egger’s regression intercept β = 0.36, SE = 1.00, *p* = 0.72). The trim-and-fill method added four hypothetical studies to the 55 observed, yielding an adjusted pooled estimate of 36.3% (95% CI: 32.4–40.3%), suggesting only minimal missing lower-yield studies. After excluding 14 studies with a high risk of bias, the sensitivity analysis of the remaining 41 studies yielded a consistent pooled estimate of 36% (95% CI: 31–40%) (Supplementary Figure 3).

Meta-regression analyses showed no significant associations between WES diagnostic yield and mean age (*n* = 30, coefficient = −0.03, *p* = 0.22), proportion of male participants (*n* = 34, coefficient = −0.01, *p* = 0.30), or publication year (*n* = 55, coefficient = 0.02, *p* = 0.49) (Supplementary Figure 4).

#### Direct comparison of CMA and WES in children with GDD/ID

**Figure 4.**
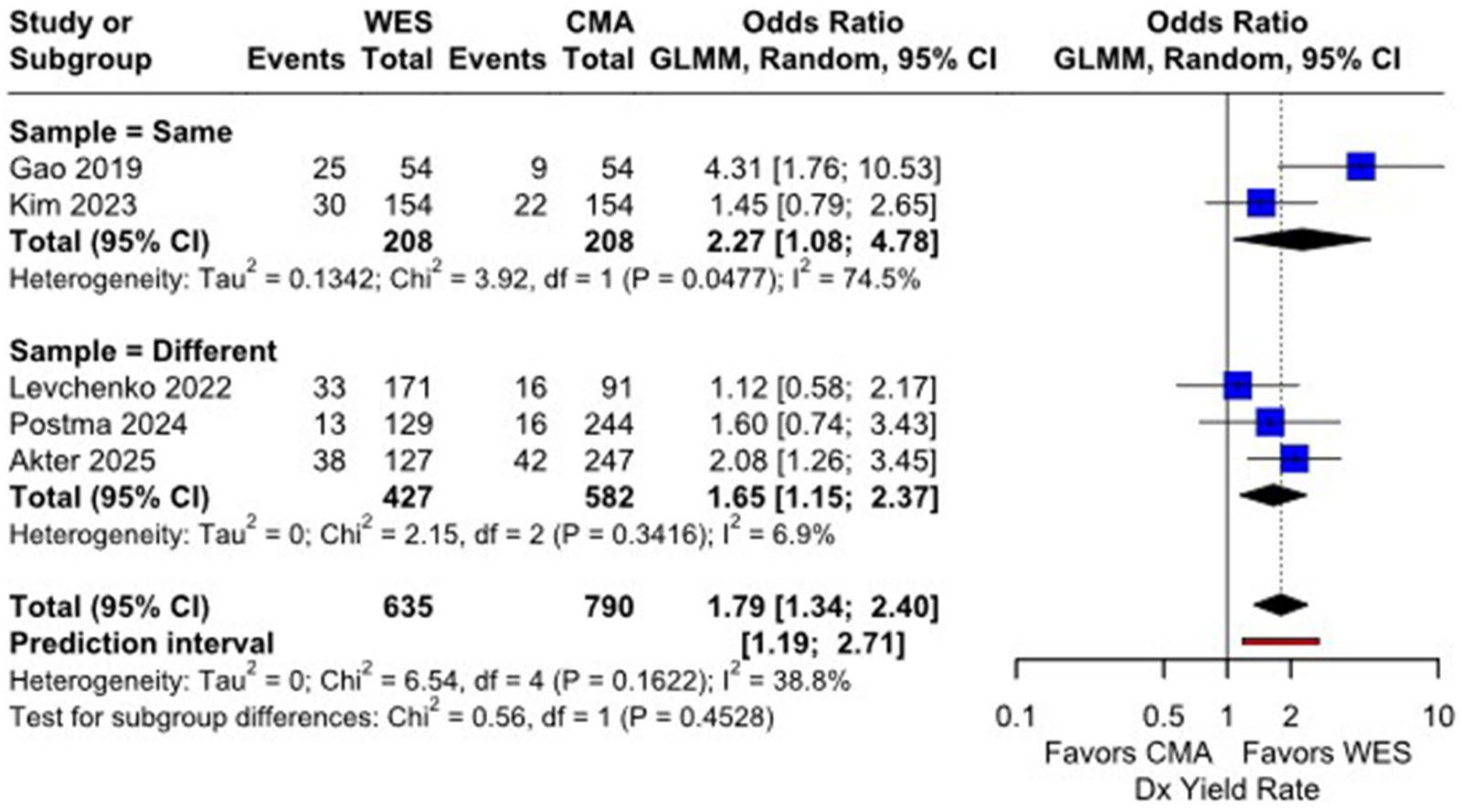
Forest plot of the proportion meta-analysis comparing the diagnostic yields between CMA and WES. CMA, chromosomal microarray; WES, whole exome sequencing.

Across five comparative cohorts (635 individuals tested by ES; 790 by CMA), WES demonstrated a significantly higher diagnostic yield than CMA. The pooled odds ratio (OR) for obtaining a diagnosis with WES versus CMA was 1.79 (95% CI: 1.34–2.40) (Figure 4). Subgroup analyses showed that WES yielded significantly higher diagnostic rates than CMA in both same-sample comparisons (OR = 2.27, 95% CI: 1.08–4.78, I^2^ = 74.5%) and different-sample comparisons (OR = 1.65, 95% CI: 1.15–2.37, I^2^ = 6.9%).

## Discussion

Our study compared the diagnostic yield and clinical utility of CMA and WES through a systematic review of eligible studies. We found that among patients with GDD/ID, WES outperformed the currently accepted first-tier test, CMA, with diagnostic yields ranging from 33–41% for WES and 16–21% for CMA, based on 102 included studies. However, the meta-analysis revealed substantial heterogeneity (I^2^ = 93.5–94.8%). Even after excluding studies with a high risk of bias in sensitivity analyses (Supplementary Figures S1 and S2), heterogeneity remained high (I^2^ = 94–95%), likely reflecting differences in study populations, settings, and methodologies. WGS, with a diagnostic yield of 26.7%, has not yet shown a clear advantage over WES.

The diagnostic yield of CMA in our analysis was 19%, which falls within the previously reported range of 13–26% [43,55,61,118]. Subgroup analysis showed a modest 2% increase in diagnostic yield in studies reporting comorbidity status—both in participants with and without comorbidities. In contrast, studies without clearly defined comorbidity information reported a slightly lower yield of 17%. Although this variation between subgroups reached statistical significance, its clinical relevance remains uncertain, particularly given the limited reporting of comorbidity status. Moreover, the number of studies with unspecified comorbidity data may have masked the true differences, making it difficult to determine whether comorbidities influenced diagnostic yield.

In contrast, the WES group demonstrated a higher diagnostic yield of 37%, nearly double that of CMA, consistent with previously reported data ranging from 37% to 61% [4,17,49,69]. Subgroup analyses revealed minimal variation in yield across studies. Again, the absence of clearly defined comorbidity data in some studies may have limited the interpretation of these results. This pattern may indicate that when ID/GDD is the primary phenotype, the presence or absence of comorbidities has little impact on diagnostic yield.

In most studies, participants underwent only one genetic test, either CMA or WES. However, we identified five studies in which both tests were performed. In these, WES clearly outperformed CMA, with an overall odds ratio (OR) of 1.79. The difference was even more pronounced in the subgroup where the same individuals underwent both tests, showing a higher diagnostic yield with an OR of 2.27. With the decreasing cost of WES in recent years, WES has increasingly emerged as the initial genetic test for children with GDD/ID.

For WGS, only one study reported a diagnostic yield of 27% in children with ID. Other studies that performed sequential testing—such as WGS following negative results from prior genetic tests—were not included in the analysis. Compared with the pooled diagnostic yield of WES at 37%, WGS demonstrated a lower yield. Given the higher cost of WGS compared with WES, additional evidence is needed before drawing a definitive conclusion regarding the relative diagnostic performance of these two approaches.

Our results did not show that the presence of additional comorbidities increases the diagnostic yield of genetic testing—particularly WES—in children with GDD/ID. For example, one study found that patients with ID and additional major medical issues received a genetic diagnosis in nearly one-third of cases, whereas patients with ASD were less likely to have an identifiable genetic etiology [119]. Another study of patients with ASD and/or ID reported that female patients and those with additional clinical findings were more likely to receive a positive result (Bishay 2017). However, our analysis focused primarily on GDD/ID populations. Neither participant age nor publication year correlated with the diagnostic yield of either test. Interestingly, meta-regression revealed that male sex was associated with a lower diagnostic yield in the CMA group. GDD and ID are known to exhibit sex bias, with an estimated male-to-female ratio of approximately 2:1. Although several studies have explored molecular and cellular sex-specific mechanisms, the results remain inconclusive [120–122].

This meta-analysis has several limitations that warrant cautious interpretation. First, the overall and subgroup data showed substantial heterogeneity (I^2^ > 90%), suggesting that comorbid conditions—such as other neurodevelopmental disorders, congenital anomalies, or neurological diseases—may not fully explain this variability. Second, the lack of standardized terminology in the field may also complicate literature searches; nonetheless, we adhered closely to our inclusion criteria, selecting only studies involving participants under 18 years of age. Third, subgroup analyses were less informative than anticipated due to limited reporting of participant characteristics in many studies. Fourth, excluding studies that performed sequential genetic testing (e.g. WES following a negative CMA result) did not improve data homogeneity. Fifth, the inclusion of both prospective and retrospective studies with varying sample sizes, while broadening the scope of the review, may have contributed to the observed heterogeneity. Finally, publication bias analysis for CMA testing suggested the presence of several missing studies, which could have led to underestimation of the diagnostic yield for CMA. In contrast, the risk of publication bias for ES testing appeared to be much lower.

GDD and ID are estimated to have a genetic etiology in up to 50% of cases. Among these, approximately 15% present with recognizable syndromic features, although some syndromes may be challenging to detect clinically [123]. Our systematic review underscores the superior diagnostic performance of ES over conventional CMA in cases of unexplained GDD/ID.

## Supplementary Material

suppl meta gen testing final.docx

PRISMA checklist meta gen.docx

## Data Availability

Data are available from authors upon request (Prof. Manit Srisurapanont: manit.s@cmu.ac.th).
