# Supplementary material for: Diagnostic and clinical utility of exome sequencing and chromosomal microarray in children with GDD/iD: a meta-analysis
Source: Ann Med. 2025 Dec 30;58(1):2609424. doi: 10.1080/07853890.2025.2609424 (PMC12777888; doi:10.1080/07853890.2025.2609424)
Supplement: suppl meta gen testing final.docx [file IANN_A_2609424_SM0637.docx]

**Supplementary Information**

**Diagnostic and clinical utility of exome sequencing and chromosomal microarray in children with GDD/ID: a meta-analysis.**

Tengsujaritkul M^1^, Louthrenoo O^1^, Likhitweerawong N^1^, Boonchooduang N^1^, Srisurapanont M^2^.

^1^Department of Pediatrics, Faculty of Medicine, Chiang Mai University, Chiang Mai, 50200 Thailand.

^2^Department of Psychiatry, Faculty of Medicine, Chiang Mai University, Chiang Mai, 50200 Thailand.

**Table of Contents**

|  | **Page** |
| --- | --- |
| Table S1. Search strategy | 2 |
| Table S2. Countries and their numbers of studies | 3 |
| Table S3. Risk of bias assessment (Hoy et al., 2012) | 4-6 |
| Fig S1. Publication bias analysis using Funnel plots and the Trim-and-Fill method for the diagnostic utility of CMA and WES | 7 |
| Fig S2. Forest plot of the sensitivity analysis for the proportion meta-analysis of CMA diagnostic rates, conducted by excluding studies with a high risk of bias | 8 |
| Fig S3. Forest plot of the sensitivity analysis for the proportion meta-analysis of WES diagnostic rates, conducted by excluding studies with a high risk of bias | 9 |
| Fig S4. Meta-regression plots depicting the association between potential moderators--mean age, percentage of male participants, and publication year—and the diagnostic yield of CMA and WES | 10 |

**Table S1.** Search strategy.

Pubmed

| **Search** | **Query** | **Results** |
| --- | --- | --- |
| #1 | (chromosomal microarray[Title/Abstract]) OR (exome sequencing[Title/Abstract]) OR (genome sequencing[Title/Abstract]) | 82,145 |
| #2 | (diagnosis[Title/Abstract]) OR clinical[Title/Abstract]) | 6,335,757 |
| #3 | (neurodevelopmental disorder[Title/Abstract]) OR (intellectual disability[Title/Abstract])) OR (global developmental delay[Title/Abstract])) | 34,759 |
| #4 | ((chromosomal microarray) OR (exome sequencing) OR (genome sequencing)) AND (diagnosis or clinical) AND ((neurodevelopmental disorder) OR (intellectual disability) OR (global developmental delay)) | 2,509 |

Scopus

| **Search** | **Query** | **Results** |
| --- | --- | --- |
| #1 | 'chromosomal microarray' OR 'exome sequencing' OR 'whole genome sequencing' | 40,906 |
| #2 | diagnosis OR clinical | 26,917,416 |
| #3 | ‘neurodevelopmental disorder’ OR ‘developmental delay’ | 46,980 |
| #4 | 'chromosomal microarray' OR 'exome sequencing' OR 'whole genome sequencing' AND (diagnosis OR clinical) AND (neurodevelopmental disorder OR developmental delay) | 2,335 |

EMBASE

| **Search** | **Query** | **Results** |
| --- | --- | --- |
| #1 | 'chromosomal microarray':ab OR 'exome sequencing':ab OR 'whole genome sequencing':ab | 90,724 |
| #2 | diagnosis:ab OR clinical:ab | 7,452,044 |
| #3 | 'neurodevelopmental disorder':ab OR 'developmental delay':ab | 35,051 |
| #4 | 'chromosomal microarray' OR 'exome sequencing' OR 'whole genome sequencing' AND (diagnosis OR clinical) AND (neurodevelopmental disorder OR developmental delay) | 3,183 |

**Table S2.** Countries and their numbers of studies in 102 included studies.

Australia 2

Bangladesh 2

Banglesh 1

Brazil 4

Bulgaria 1

Canada 2

China 23

Croatia 1

Czech Republic 1

Germany 2

India 5

Iran 3

Israel 1

Italy 5

Japan 2

Jordan 1

Korea 10

Lebanon 1

Lithuania 2

Netherlands 3

Peru 1

Poland 2

Romania 1

Russia 3

Serbia 1

Singapore 2

South Africa and Kenya 1

Spain 6

Switzerland 1

Taiwan 1

The Netherlands 2

The United Arab Emirates 1

Turkey 6

USA 7

USA and Canada 1

Ukraine 1

**Table S3.** Risk of bias assessment of included studies.

| Study | Item and scoring (low risk = 0, high risk = 1) | | | | | | | | | | Total score |  |
| --- | --- | --- | --- | --- | --- | --- | --- | --- | --- | --- | --- | --- |
|  | 1 | 2 | 3 | 4 | 5 | 6 | 7 | 8 | 9 | 10 | 11 |  |
| Abarca-Barriga 2025 | 0 | 0 | 0 | 1 | 0 | 1 | 0 | 0 | 0 | 0 | 2 | low |
| Abe-Hatano 2021 | 1 | 0 | 0 | 1 | 0 | 1 | 0 | 0 | 0 | 0 | 3 | low |
| Akkus 2024 | 0 | 0 | 0 | 1 | 0 | 1 | 0 | 0 | 1 | 1 | 4 | mod |
| Akter 2023 | 0 | 0 | 1 | 1 | 0 | 1 | 0 | 0 | 1 | 1 | 5 | mod |
| Akter 2025 | 0 | 0 | 0 | 1 | 0 | 1 | 0 | 0 | 0 | 0 | 2 | low |
| Arican 2018 | 1 | 1 | 1 | 1 | 0 | 1 | 0 | 1 | 1 | 1 | 8 | high |
| Asadollahi 2014 | 1 | 1 | 1 | 1 | 0 | 1 | 0 | 0 | 1 | 1 | 7 | high |
| Ballesta-Martínez 2023 | 0 | 0 | 0 | 1 | 0 | 1 | 0 | 0 | 0 | 1 | 3 | low |
| Bartnik 2014 | 1 | 1 | 1 | 1 | 0 | 1 | 0 | 0 | 1 | 1 | 7 | high |
| Battaglia 2013 | 0 | 0 | 0 | 1 | 0 | 1 | 0 | 0 | 0 | 0 | 2 | low |
| Bhatia 2021 | 0 | 0 | 0 | 1 | 0 | 1 | 0 | 0 | 0 | 1 | 3 | low |
| Boyarchuk 2024 | 0 | 0 | 0 | 1 | 0 | 1 | 0 | 0 | 0 | 0 | 2 | low |
| Brea-Fernández 2022 | 0 | 0 | 0 | 1 | 0 | 0 | 0 | 0 | 1 | 0 | 2 | low |
| Çebi 2020 | 0 | 0 | 0 | 1 | 0 | 1 | 0 | 0 | 0 | 0 | 2 | low |
| Cham 2017 | 1 | 1 | 1 | 1 | 0 | 1 | 0 | 1 | 1 | 1 | 8 | high |
| Chaves 2019 | 0 | 0 | 0 | 1 | 0 | 0 | 0 | 0 | 0 | 0 | 1 | low |
| Chaves 2024 | 0 | 0 | 0 | 1 | 0 | 0 | 0 | 0 | 0 | 0 | 1 | low |
| Chen 2021 | 1 | 1 | 1 | 1 | 0 | 1 | 0 | 1 | 1 | 1 | 8 | high |
| Choucair 2015 | 1 | 1 | 1 | 1 | 0 | 1 | 0 | 1 | 1 | 1 | 8 | high |
| Cooper 2011 | 0 | 0 | 0 | 1 | 0 | 1 | 0 | 0 | 1 | 1 | 4 | mod |
| de Ligt 2012 | 0 | 0 | 0 | 1 | 0 | 1 | 0 | 0 | 1 | 1 | 4 | mod |
| de Souza 2019 | 0 | 0 | 1 | 1 | 0 | 1 | 0 | 0 | 0 | 0 | 3 | low |
| Di Biagio 2023 | 1 | 1 | 1 | 1 | 0 | 1 | 0 | 1 | 1 | 1 | 8 | high |
| Di Gregorio 2017 | 0 | 0 | 0 | 1 | 0 | 1 | 0 | 0 | 1 | 1 | 4 | mod |
| Dingemans 2022 | 0 | 0 | 0 | 1 | 0 | 1 | 0 | 0 | 1 | 0 | 3 | low |
| Dong 2020 | 0 | 0 | 0 | 1 | 0 | 1 | 0 | 0 | 0 | 0 | 2 | low |
| Ezugha 2010 | 1 | 1 | 1 | 1 | 0 | 1 | 0 | 0 | 0 | 1 | 6 | mod |
| Gao 2019 | 1 | 1 | 0 | 1 | 0 | 1 | 0 | 0 | 0 | 0 | 4 | mod |
| Ghalamkari 2025 | 0 | 0 | 0 | 1 | 0 | 1 | 0 | 0 | 1 | 1 | 4 | mod |
| Girirajan 2011 | 0 | 0 | 0 | 1 | 0 | 0 | 0 | 0 | 1 | 1 | 3 | low |
| Gürkan 2020 | 0 | 0 | 0 | 1 | 0 | 0 | 0 | 0 | 1 | 1 | 3 | low |
| Henderson 2014 | 1 | 1 | 0 | 1 | 0 | 1 | 0 | 0 | 0 | 1 | 5 | mod |
| Hu 2019 | 1 | 1 | 1 | 1 | 0 | 1 | 0 | 1 | 1 | 1 | 8 | high |
| Hu 2017 | 0 | 0 | 0 | 1 | 0 | 0 | 0 | 0 | 0 | 1 | 2 | low |
| Ilic 2024 | 0 | 0 | 0 | 1 | 0 | 1 | 0 | 0 | 0 | 0 | 2 | low |
| Jo 2024 | 0 | 0 | 1 | 1 | 0 | 0 | 0 | 0 | 0 | 0 | 2 | low |
| Kamath 2022 | 0 | 1 | 1 | 1 | 0 | 1 | 0 | 0 | 0 | 1 | 5 | mod |
| Kanivets 2017 | 1 | 1 | 1 | 1 | 0 | 1 | 0 | 1 | 1 | 1 | 8 | high |
| Khalaf 2024 | 0 | 1 | 1 | 1 | 0 | 1 | 0 | 0 | 0 | 1 | 5 | mod |
| Kim 2018 | 0 | 0 | 1 | 1 | 0 | 0 | 0 | 0 | 0 | 0 | 2 | low |
| Kim 2023 | 1 | 1 | 1 | 1 | 0 | 1 | 0 | 1 | 1 | 1 | 8 | high |
| Kim 2019 | 0 | 0 | 0 | 1 | 0 | 0 | 0 | 0 | 0 | 0 | 1 | low |
| Kipkemoi 2023 | 0 | 0 | 0 | 1 | 0 | 1 | 0 | 0 | 1 | 1 | 4 | mod |
| Lai G 2024 | 0 | 0 | 0 | 1 | 0 | 1 | 0 | 0 | 0 | 0 | 2 | low |
| Lamilla 2025 | 0 | 0 | 0 | 1 | 0 | 0 | 0 | 0 | 0 | 0 | 1 | low |
| Lan 2025 | 0 | 0 | 0 | 1 | 0 | 1 | 0 | 0 | 0 | 0 | 2 | low |
| Lee 2019 | 1 | 1 | 1 | 1 | 0 | 1 | 0 | 1 | 1 | 1 | 8 | high |
| Lee 2018 | 0 | 0 | 0 | 1 | 0 | 1 | 0 | 0 | 0 | 0 | 2 | low |
| Lee 2017 | 0 | 0 | 0 | 1 | 0 | 1 | 0 | 0 | 0 | 0 | 2 | low |
| Levchenko 2022 | 0 | 1 | 0 | 1 | 0 | 1 | 0 | 0 | 0 | 0 | 3 | low |
| Li 2024 | 0 | 0 | 0 | 1 | 0 | 0 | 0 | 0 | 0 | 1 | 2 | low |
| Liu 2022 | 0 | 0 | 0 | 1 | 0 | 1 | 0 | 0 | 0 | 1 | 3 | low |
| Liu 2021 | 0 | 0 | 0 | 1 | 0 | 1 | 0 | 0 | 1 | 0 | 3 | low |
| Ma 2024 | 0 | 0 | 0 | 1 | 0 | 1 | 0 | 0 | 0 | 0 | 2 | low |
| Masri 2023 | 1 | 1 | 1 | 1 | 0 | 1 | 0 | 1 | 1 | 1 | 8 | high |
| Miclea 2022 | 0 | 0 | 0 | 1 | 0 | 1 | 0 | 0 | 0 | 0 | 2 | low |
| Monroe 2016 | 1 | 1 | 1 | 1 | 0 | 1 | 0 | 0 | 1 | 1 | 7 | high |
| Najafi 2019 | 1 | 1 | 1 | 1 | 0 | 1 | 0 | 1 | 1 | 1 | 8 | high |
| Neuhann 2021 | 1 | 1 | 1 | 1 | 0 | 1 | 0 | 1 | 1 | 1 | 8 | high |
| Newman 2016 | 1 | 1 | 1 | 1 | 0 | 1 | 0 | 1 | 1 | 1 | 8 | high |
| Nicholl 2014 | 0 | 0 | 0 | 1 | 0 | 1 | 0 | 0 | 1 | 1 | 4 | mod |
| Nouri 2021 | 1 | 1 | 1 | 1 | 0 | 1 | 0 | 1 | 1 | 1 | 8 | high |
| Nowakowska 2008 | 0 | 0 | 0 | 1 | 0 | 1 | 0 | 0 | 1 | 1 | 4 | mod |
| Oğuz 2021 | 0 | 0 | 0 | 1 | 0 | 1 | 0 | 0 | 1 | 0 | 3 | low |
| Palmer 2014 | 1 | 1 | 1 | 1 | 0 | 1 | 0 | 1 | 1 | 1 | 8 | high |
| Palmieri 2023 | 1 | 1 | 1 | 1 | 0 | 1 | 0 | 1 | 1 | 1 | 8 | high |
| Pati 2015 | 1 | 1 | 1 | 1 | 0 | 1 | 0 | 1 | 1 | 1 | 8 | high |
| Pereira 2014 | 1 | 1 | 1 | 1 | 0 | 1 | 0 | 0 | 0 | 1 | 6 | mod |
| Pérez-Granero 2017 | 1 | 1 | 1 | 1 | 0 | 1 | 0 | 1 | 1 | 1 | 8 | high |
| Peycheva 2018 | 0 | 0 | 0 | 1 | 0 | 1 | 0 | 0 | 1 | 0 | 3 | low |
| Postma 2024 | 0 | 0 | 0 | 1 | 0 | 1 | 0 | 0 | 0 | 0 | 2 | low |
| Pranav Chand 2023 | 0 | 0 | 0 | 1 | 0 | 1 | 0 | 0 | 0 | 1 | 3 | low |
| Preiksaitiene 2016 | 0 | 0 | 0 | 1 | 0 | 0 | 0 | 0 | 1 | 1 | 3 | low |
| Preiksaitiene 2014 | 1 | 0 | 0 | 1 | 0 | 1 | 0 | 0 | 1 | 1 | 5 | mod |
| Qian 2025 | 0 | 0 | 0 | 1 | 0 | 1 | 0 | 0 | 0 | 1 | 3 | low |
| Quintela 2017 | 0 | 0 | 0 | 1 | 0 | 1 | 0 | 0 | 1 | 1 | 4 | mod |
| Rauch 2012 | 0 | 1 | 1 | 1 | 0 | 0 | 0 | 0 | 1 | 1 | 5 | mod |
| Rump 2016 | 1 | 1 | 1 | 1 | 0 | 1 | 0 | 0 | 1 | 1 | 7 | high |
| Sánchez Suárez 2024 | 0 | 0 | 0 | 1 | 0 | 1 | 0 | 0 | 0 | 0 | 2 | low |
| Sandal 2024 | 1 | 1 | 1 | 1 | 0 | 1 | 0 | 1 | 1 | 1 | 8 | high |
| Sansovic 2017 | 0 | 0 | 0 | 1 | 0 | 1 | 0 | 0 | 1 | 1 | 4 | mod |
| Seo 2022 | 1 | 1 | 1 | 1 | 0 | 1 | 0 | 1 | 1 | 1 | 8 | high |
| Sharma 2016 | 0 | 1 | 1 | 1 | 0 | 1 | 0 | 0 | 0 | 0 | 4 | mod |
| Shchubelka 2024 | 0 | 0 | 0 | 1 | 0 | 0 | 0 | 0 | 1 | 0 | 2 | low |
| Shin 2015 | 0 | 0 | 0 | 1 | 0 | 1 | 0 | 0 | 0 | 0 | 2 | low |
| Stojanovic 2020 | 1 | 1 | 0 | 1 | 0 | 1 | 0 | 0 | 0 | 0 | 4 | mod |
| Taskiran 2021 | 1 | 1 | 1 | 1 | 0 | 0 | 0 | 0 | 1 | 0 | 5 | mod |
| Valaparambil 2025 | 0 | 1 | 0 | 1 | 0 | 1 | 0 | 1 | 0 | 0 | 4 | mod |
| Valentino 2021 | 0 | 0 | 0 | 1 | 0 | 1 | 0 | 0 | 0 | 0 | 2 | low |
| Vrijenhoek 2018 | 0 | 0 | 0 | 1 | 0 | 1 | 0 | 0 | 0 | 1 | 3 | low |
| Wayhelova 2024 | 0 | 0 | 0 | 1 | 0 | 1 | 0 | 0 | 1 | 1 | 4 | mod |
| Weiss 2021 | 1 | 1 | 1 | 1 | 0 | 1 | 0 | 1 | 1 | 1 | 8 | high |
| Wu 2023 | 1 | 1 | 1 | 1 | 0 | 1 | 0 | 1 | 1 | 1 | 8 | high |
| Wu 2025 | 0 | 0 | 0 | 1 | 0 | 1 | 0 | 0 | 0 | 0 | 2 | low |
| Wu 2024 | 0 | 0 | 0 | 1 | 0 | 0 | 0 | 0 | 0 | 1 | 2 | low |
| Wu 2021 | 0 | 0 | 0 | 1 | 0 | 0 | 0 | 0 | 0 | 1 | 2 | low |
| Xiang 2021 | 1 | 1 | 1 | 1 | 0 | 1 | 0 | 0 | 1 | 1 | 7 | high |
| Xiao 2018 | 1 | 1 | 1 | 1 | 0 | 1 | 0 | 0 | 1 | 1 | 7 | high |
| Xu 2024 | 0 | 0 | 0 | 1 | 0 | 1 | 0 | 0 | 0 | 0 | 2 | low |
| Yamamoto 2019 | 0 | 0 | 0 | 1 | 0 | 0 | 0 | 0 | 1 | 1 | 3 | low |
| Yuan 2021 | 0 | 0 | 0 | 1 | 0 | 1 | 0 | 0 | 1 | 0 | 3 | low |
| Zhang 2024 | 0 | 0 | 0 | 1 | 0 | 1 | 0 | 0 | 0 | 0 | 2 | low |

**Tool for assessing risk of bias in prevalence studies***

External validity

1. Was the study’s target population a close representation of the national population in relation to relevant variables?

2. Was the sampling frame a true or close representation of the target population?

3. Was some form of random selection used to select the sample, OR was a census undertaken?

4. Was the likelihood of nonresponse bias minimal?

Internal validity

5. Were data collected directly from the subjects (as opposed to a proxy)?

6. Was an acceptable case definition used in the study?

7. Was the study instrument that measured the parameter of interest shown to have validity and reliability?

8. Was the same mode of data collection used for all subjects?

9. Was the length of the shortest prevalence period for the parameter of interest appropriate?

10. Were the numerator(s) and denominator(s) for the parameter of interest appropriate?

11. Summary item on the overall risk of study bias

Overall risk based on total point: low risk (0-3), moderate risk (4-6), and high risk (7-9).

* Hoy D, Brooks P, Woolf A, Blyth F, March L, Bain C, Baker P, Smith E, Buchbinder R. Assessing risk of bias in prevalence studies: modification of an existing tool and evidence of interrater agreement. J. Clin. Epidemiol. 2012;65: 934–939.

| A.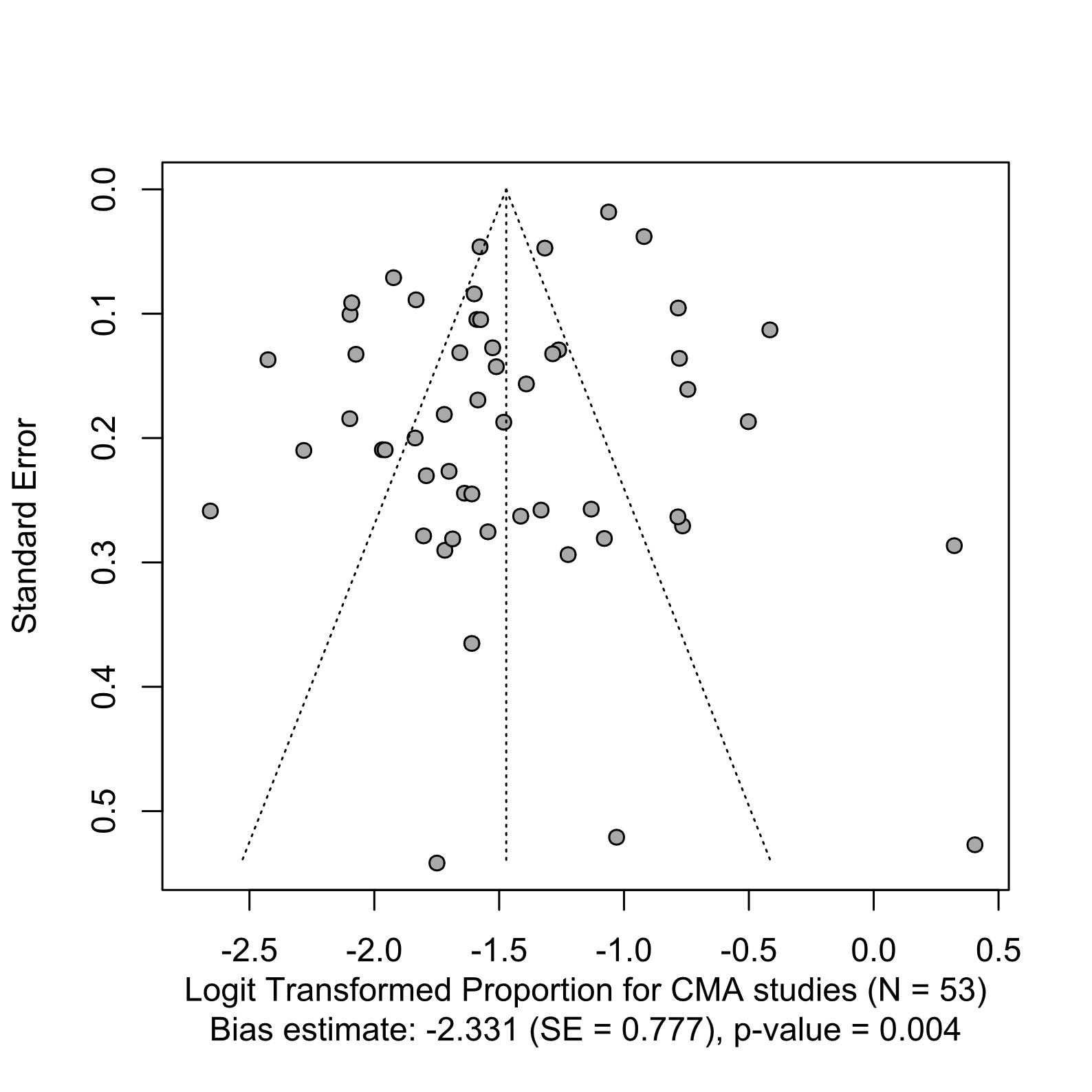 | B.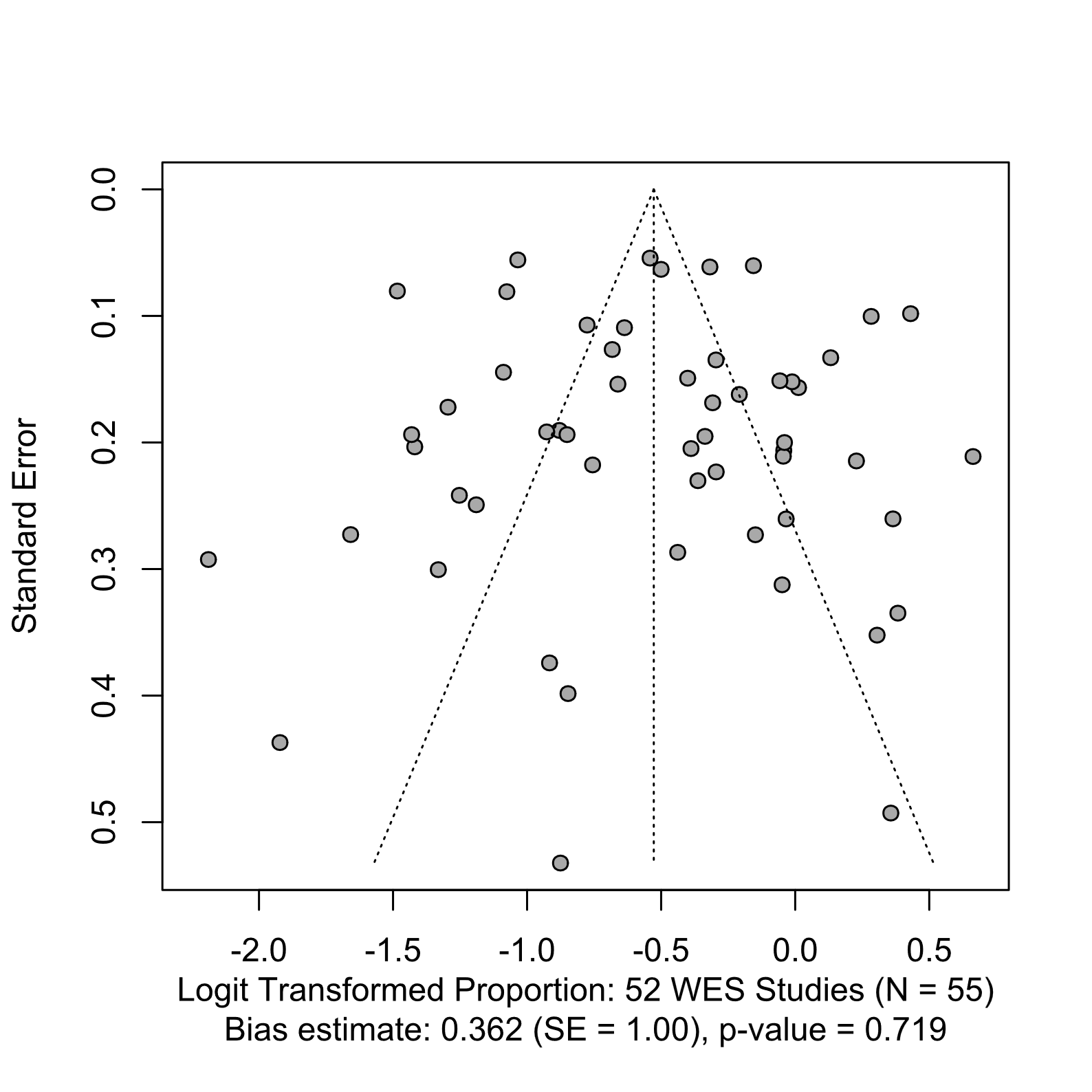 |
| --- | --- |
| C.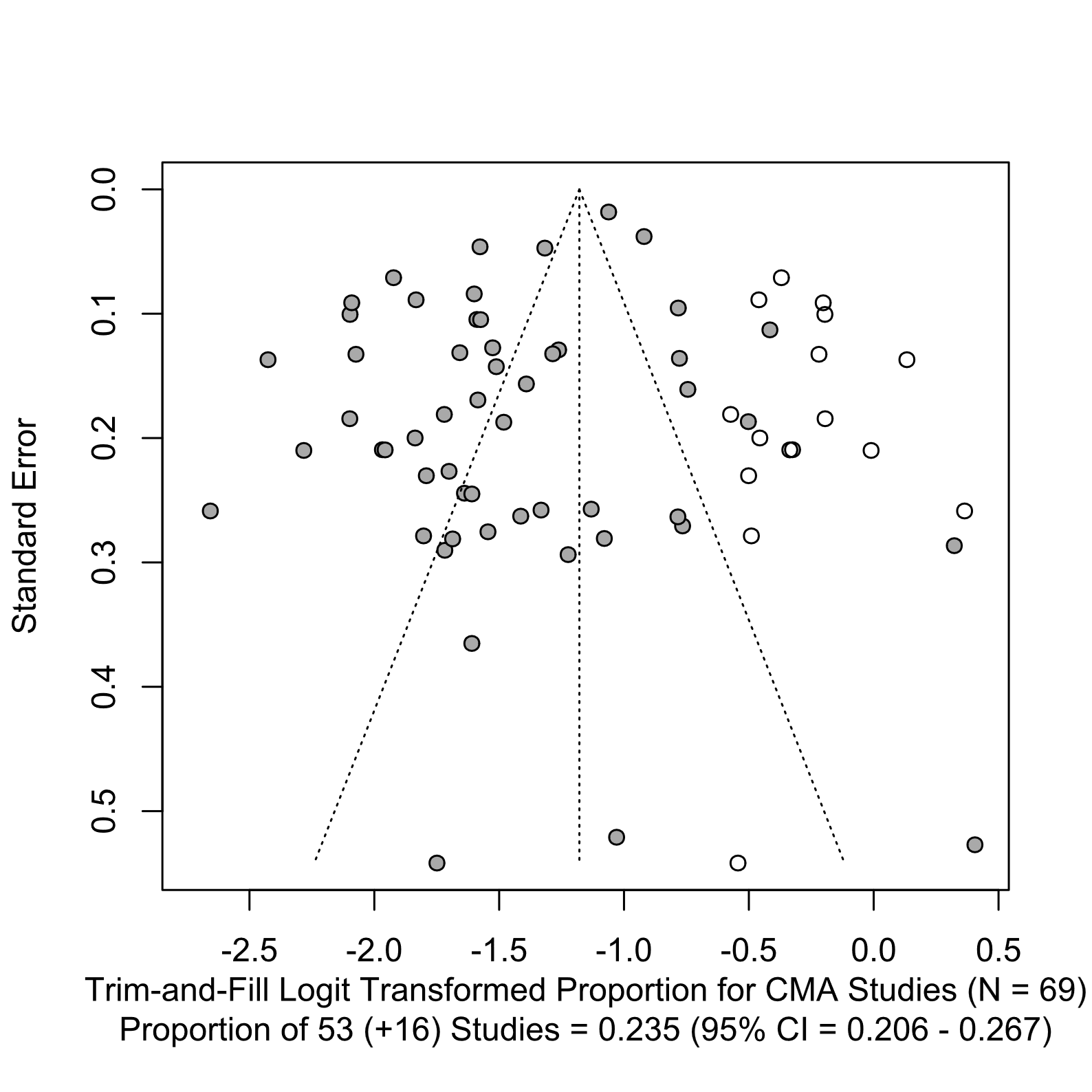 | D.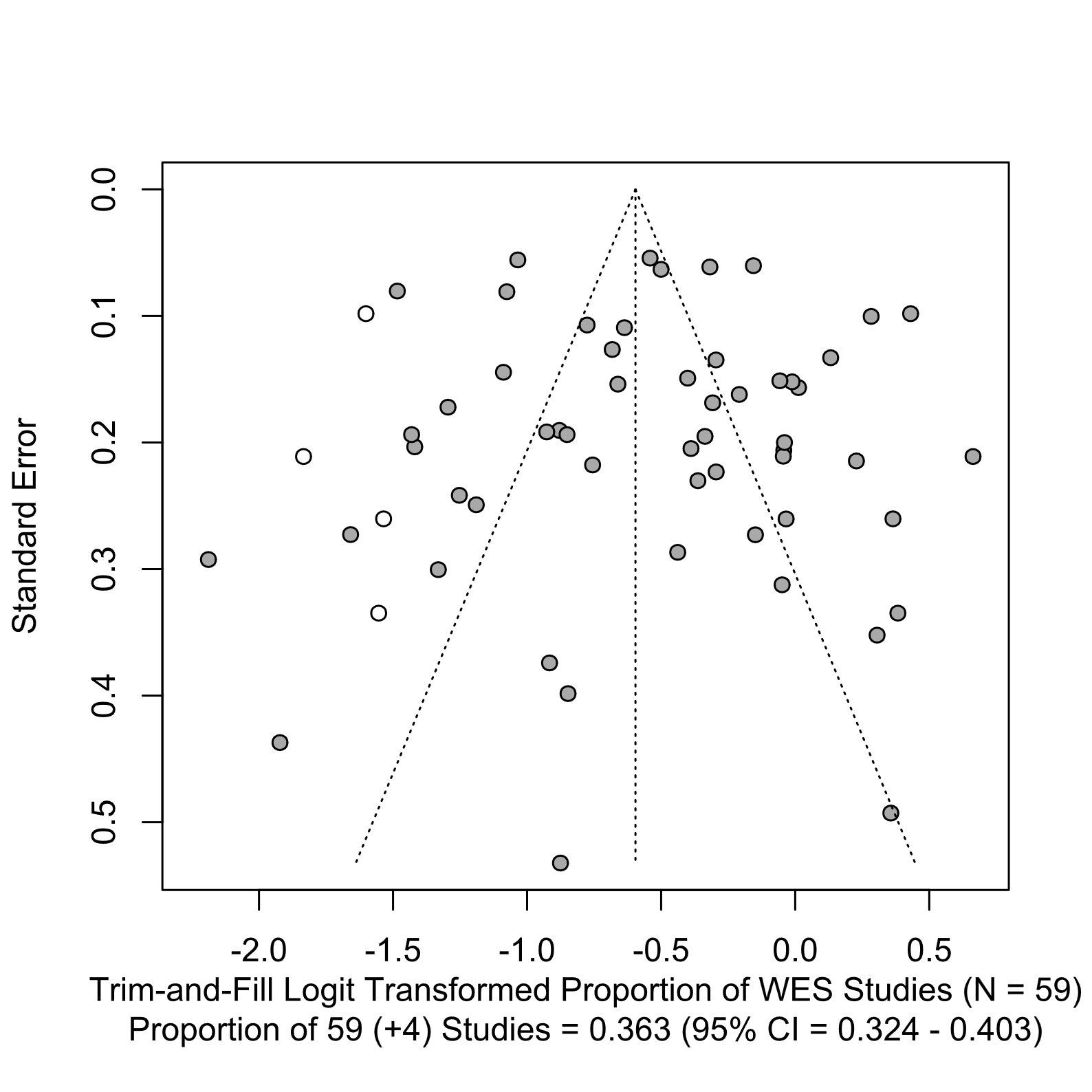 |

**Fig S1.** Publication bias analysis using Funnel plots (A-B) and the Trim-and-Fill method (C-D) for the diagnostic utility of CMA and WES.


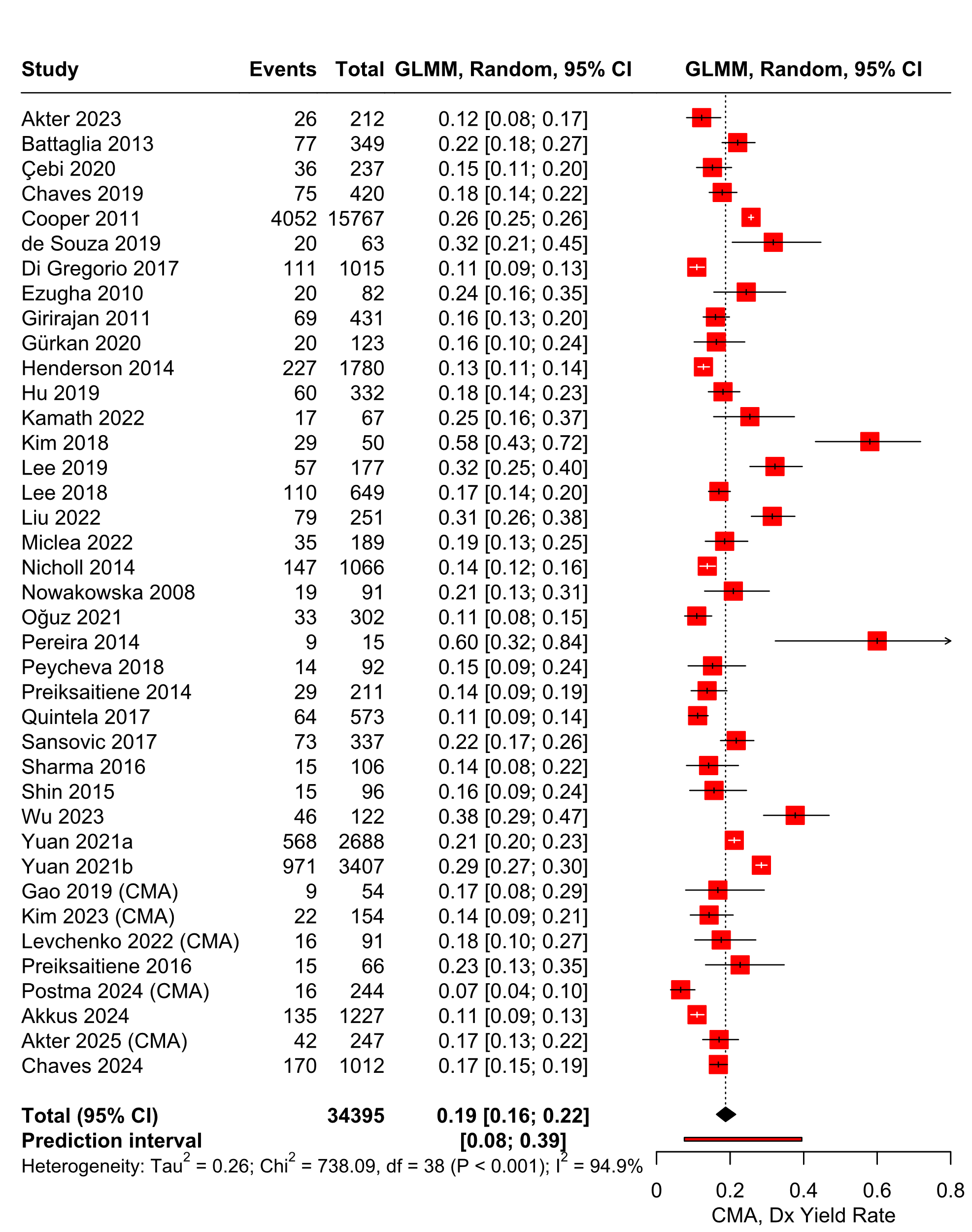


**Fig S2.** Forest plot of the sensitivity analysis for the proportion meta-analysis of CMA diagnostic rates, conducted by excluding studies with a high risk of bias.


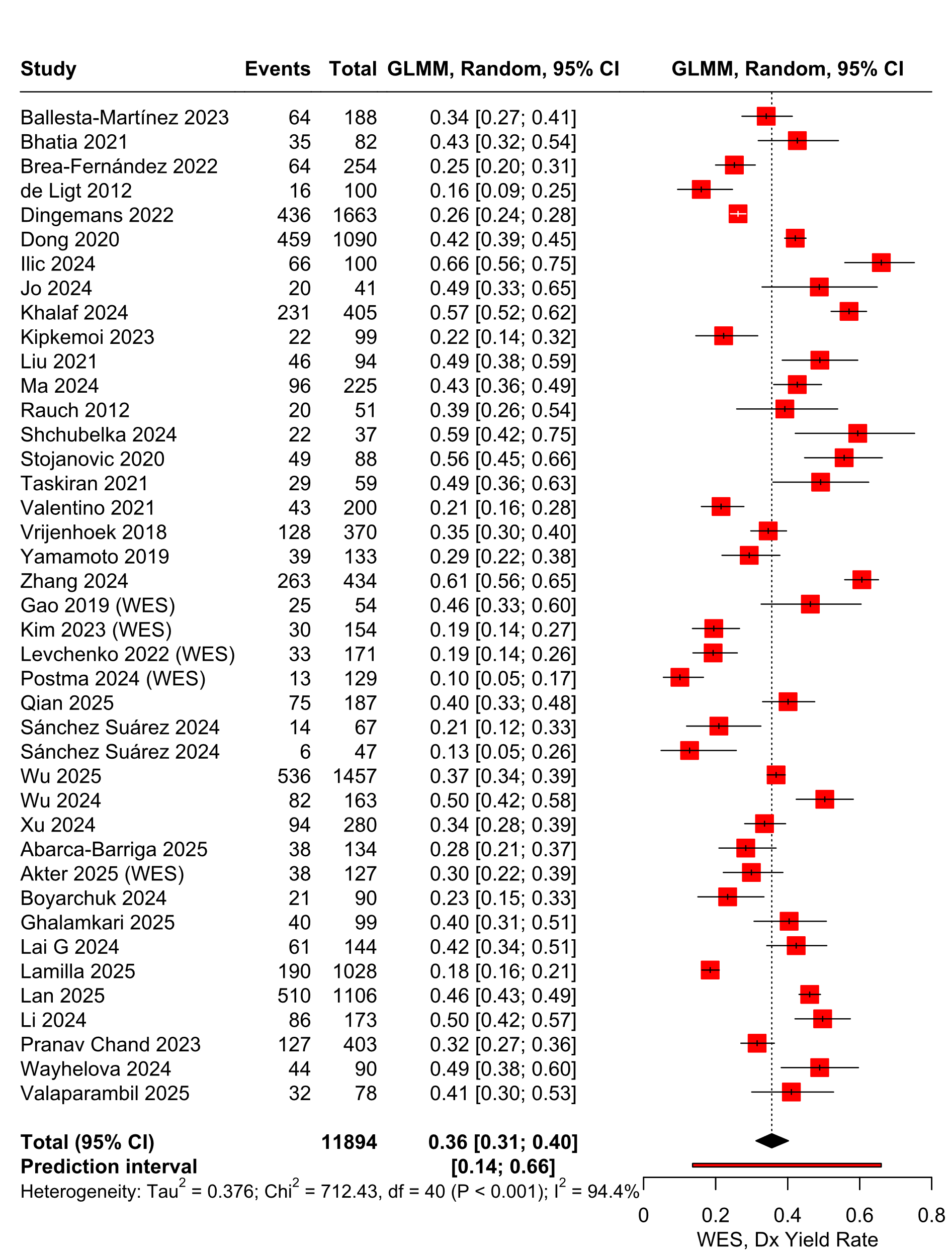


**Fig S3.** Forest plot of the sensitivity analysis for the proportion meta-analysis of WES diagnostic rates, conducted by excluding studies with a high risk of bias.

| A.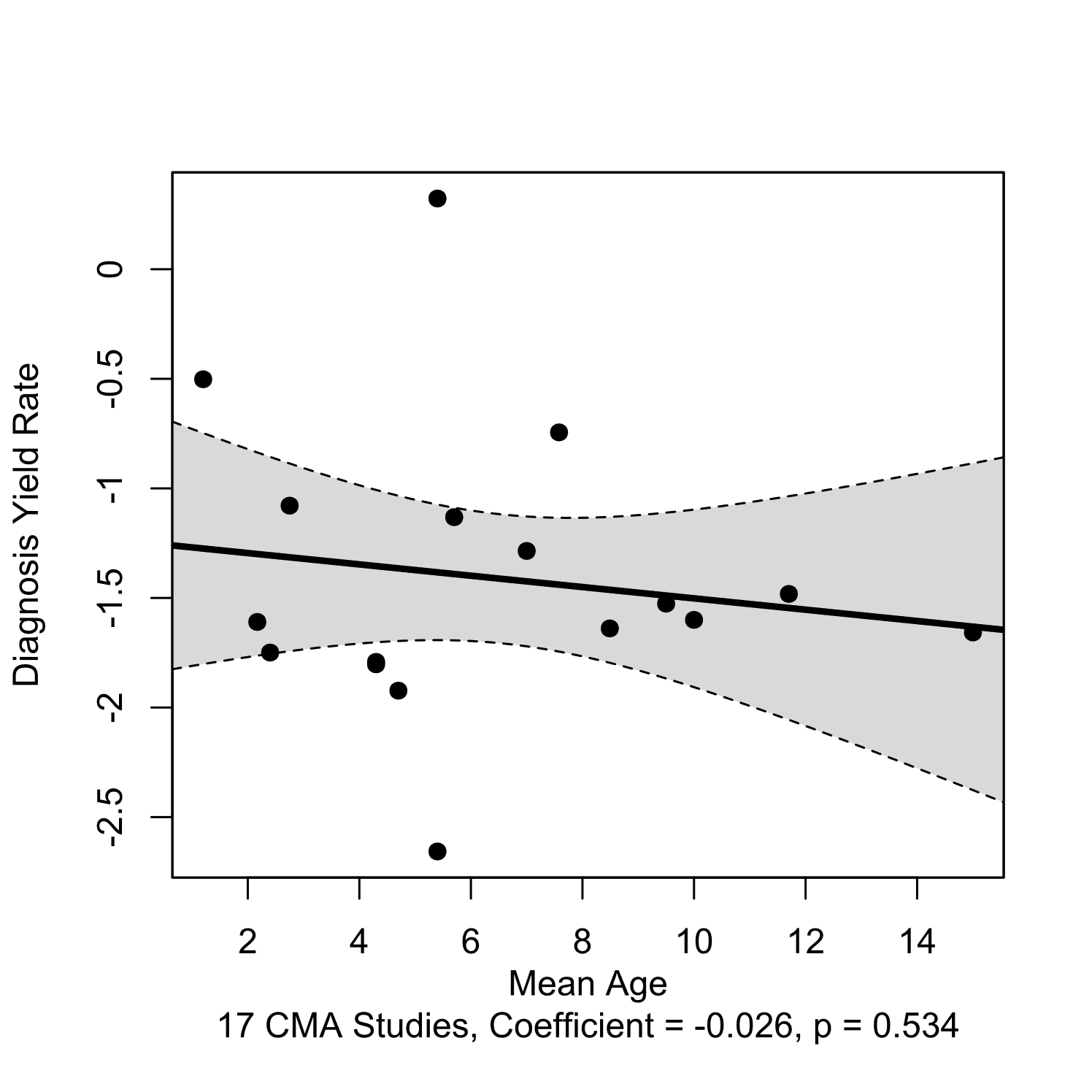 | B.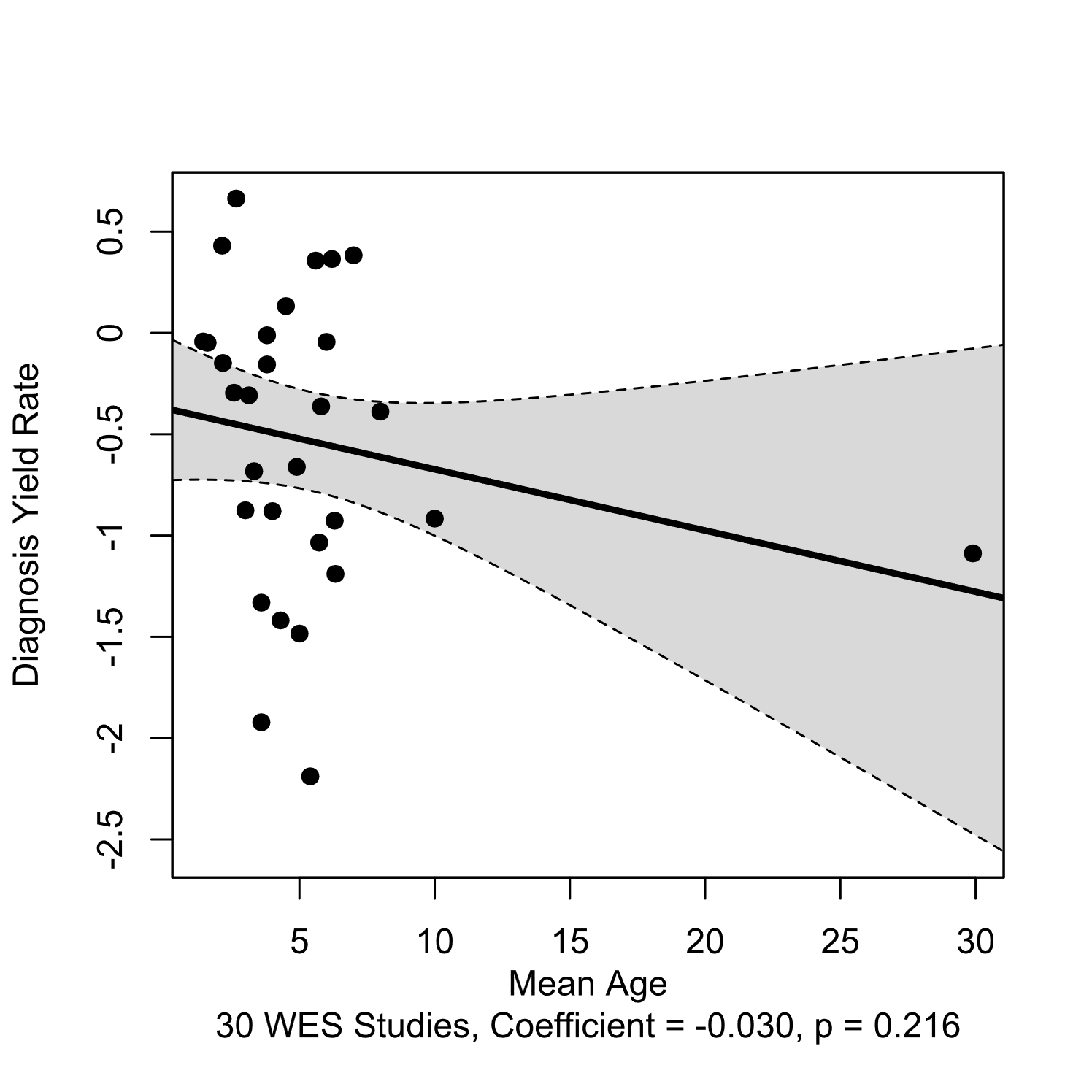 |
| --- | --- |
| C.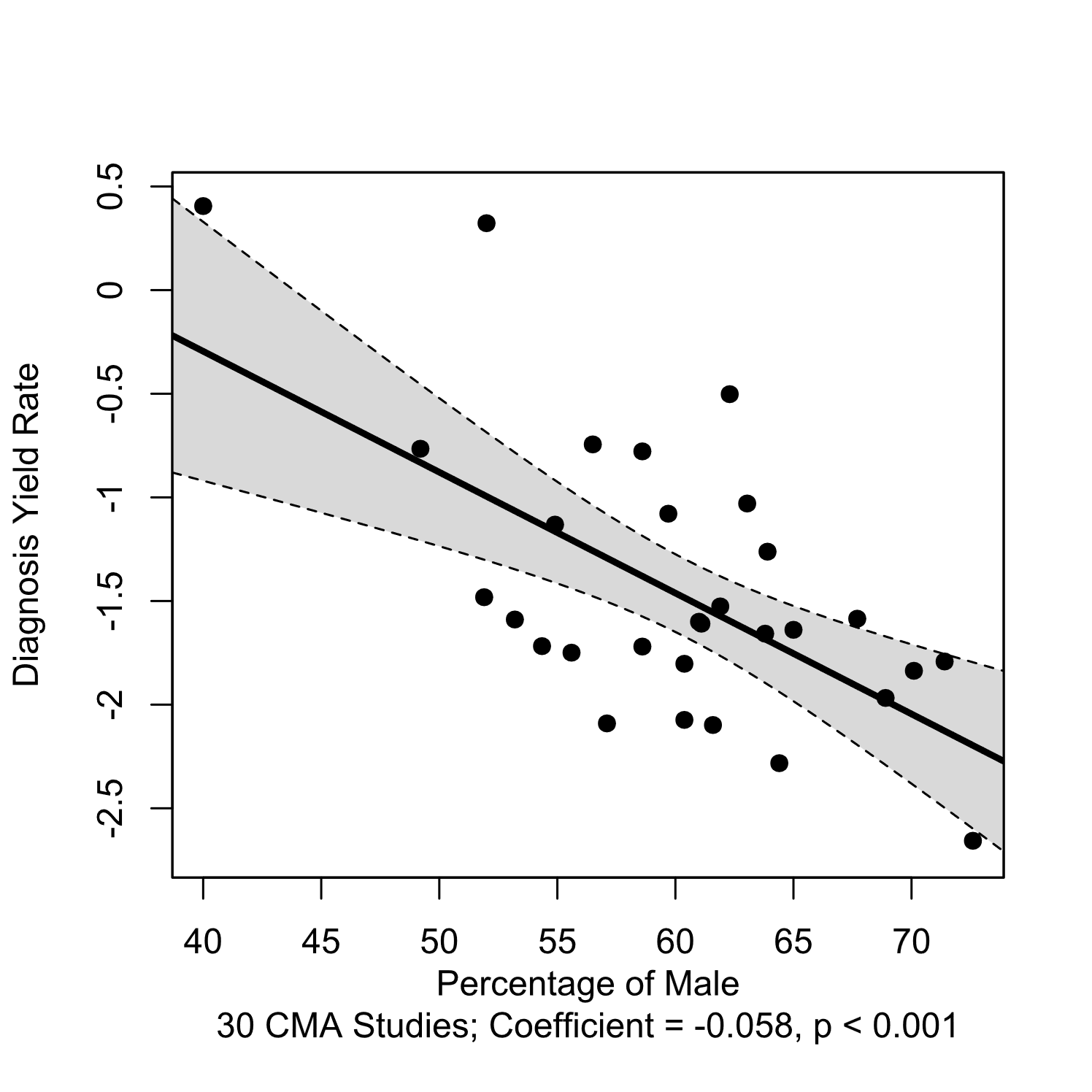 | D.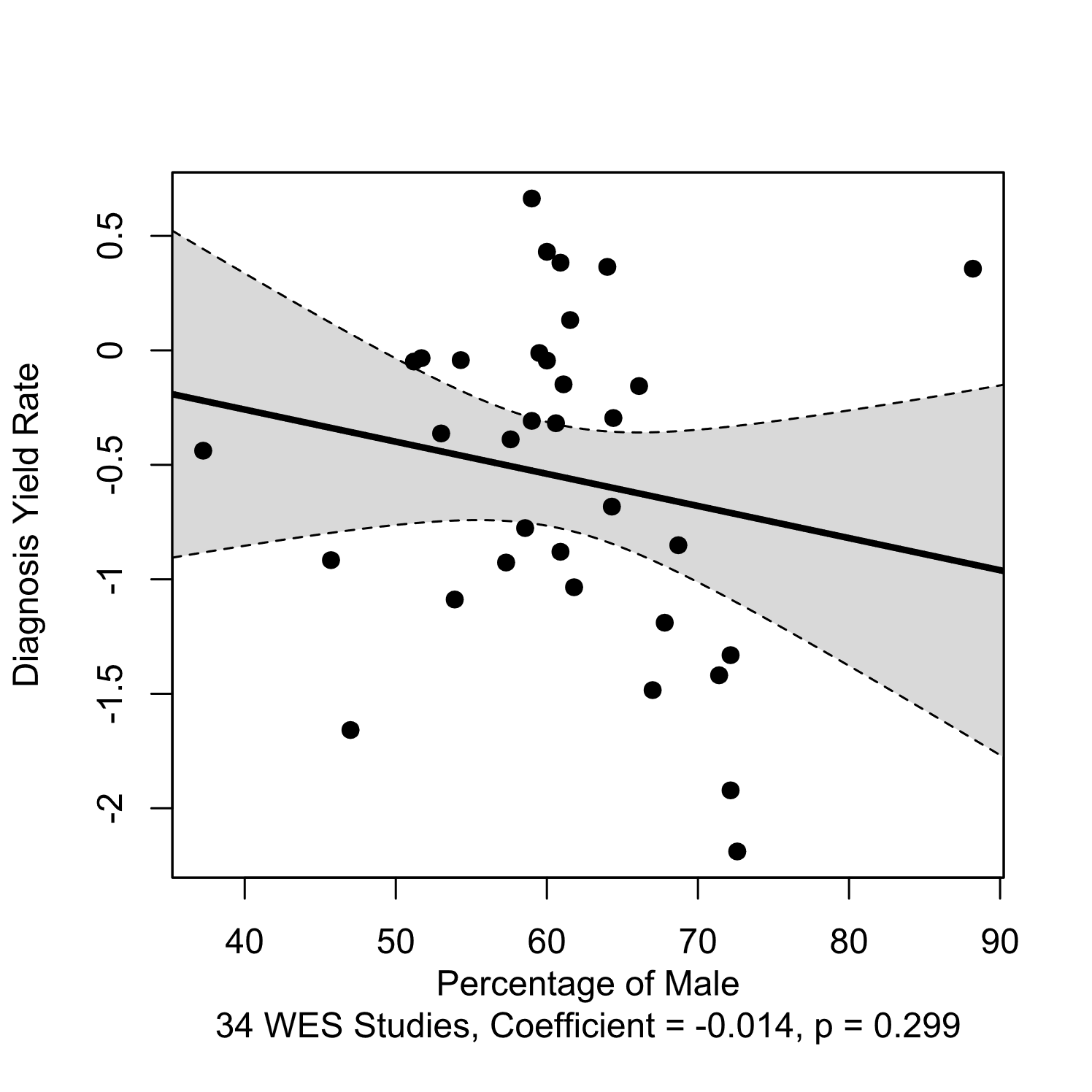 |
| E.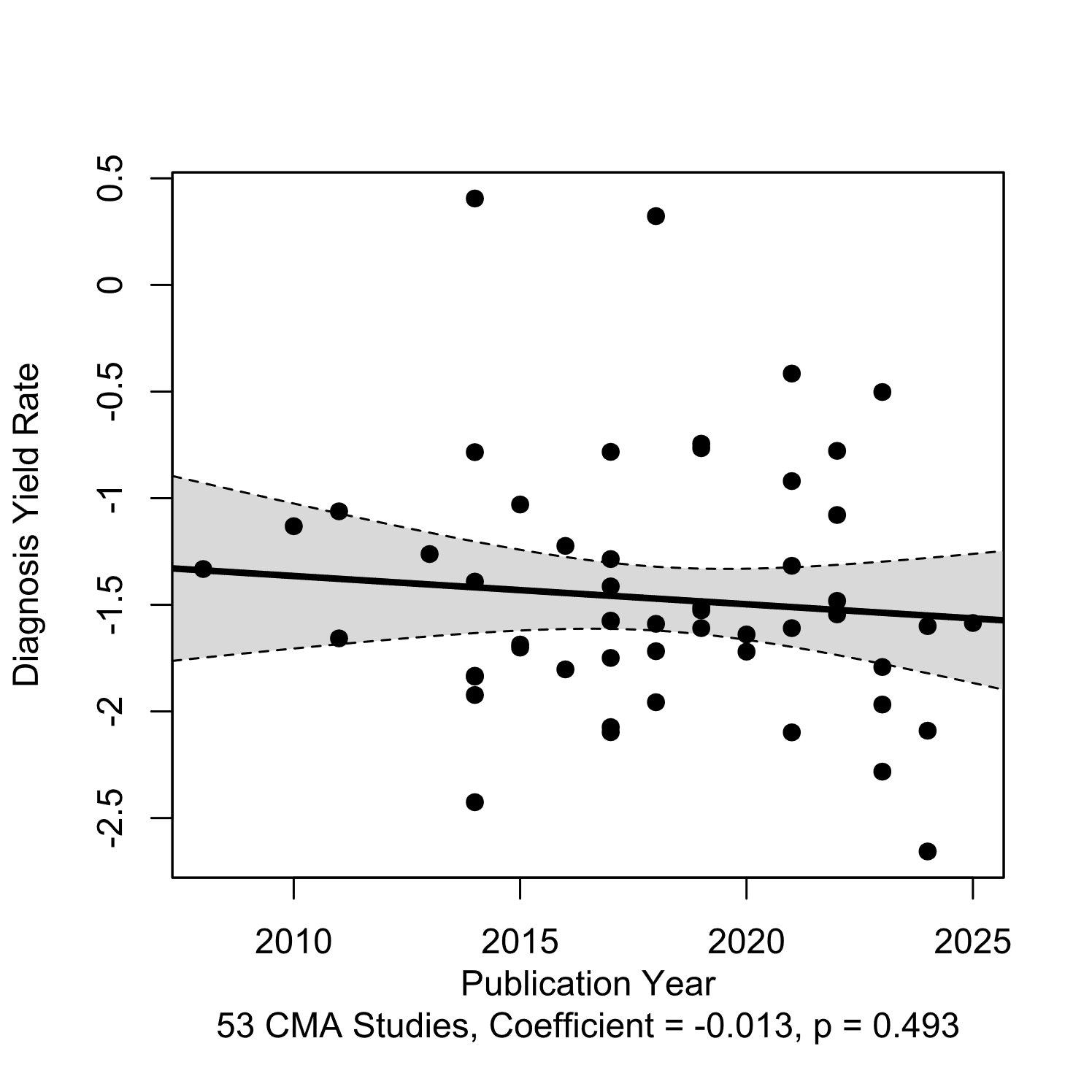 | F.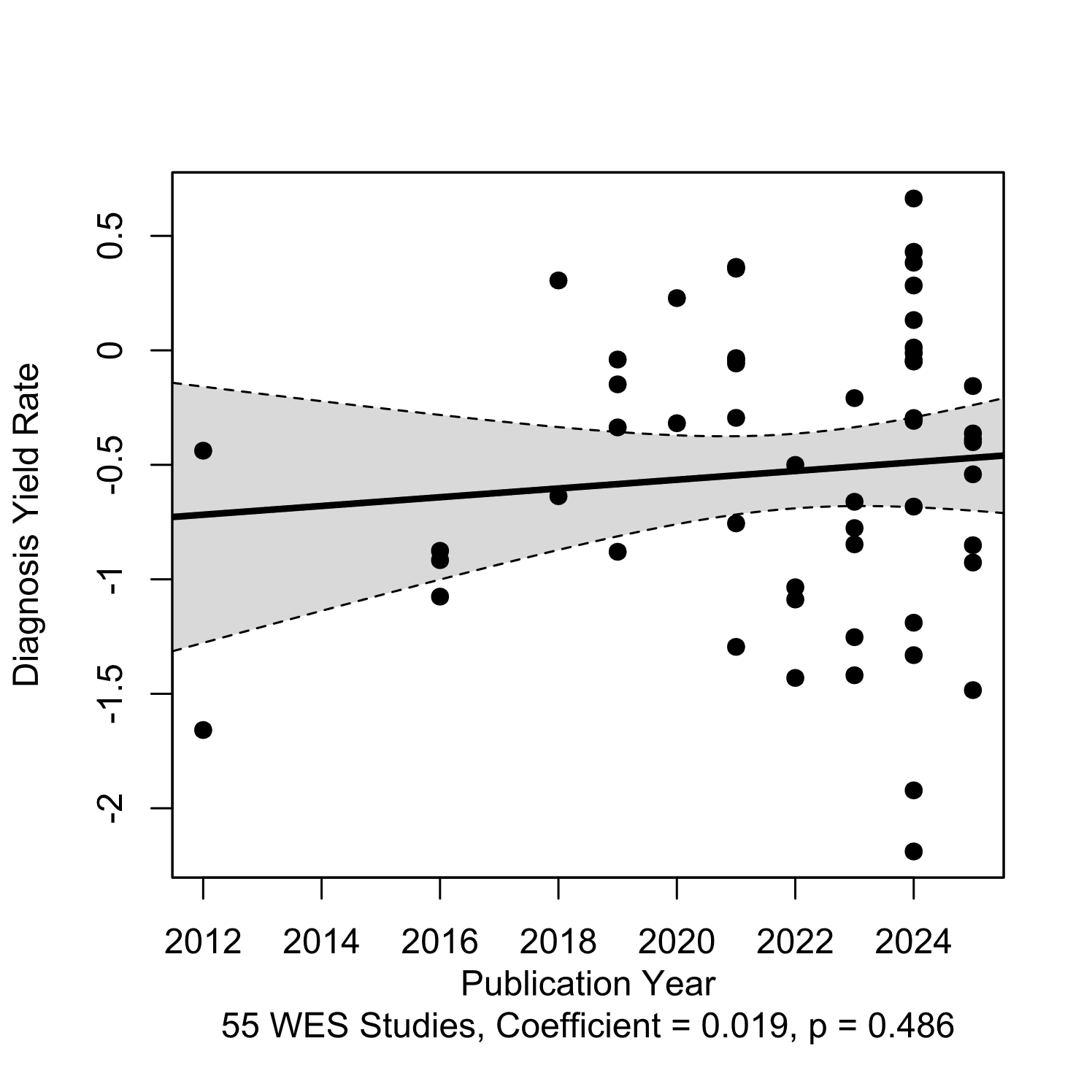 |

**Fig S4.** Meta-regression plots depicting the association between potential moderators--mean age (A-B), percentage of male participants (C-D), and publication year of the study (E-F)—and the diagnostic yield of CMA and WES.
